# The effect of infection with the entomopathogenic fungus *Conidiobolus coronatus* (Entomopthorales) on eighteen cytokine-like proteins in *Galleria mellonella* (Lepidoptera) larvae

**DOI:** 10.3389/fimmu.2024.1385863

**Published:** 2024-05-07

**Authors:** Anna Katarzyna Wrońska, Agata Kaczmarek, Justyna Sobich, Mieczysława Irena Boguś

**Affiliations:** ^1^ Museum and Institute of Zoology, Polish Academy of Science, Warsaw, Poland; ^2^ Dioscuri Centre for RNA-Protein Interactions in Human Health and Disease, International Institute of Molecular and Cell Biology in Warsaw, Warsaw, Poland

**Keywords:** cytokines, insects hemocytes, fungal infection, model in immunological research, *Galleria mellollella*, *Conidiobolus coronatus*

## Abstract

**Background:**

In response to the replace mammal research models with insects in preliminary immunological studies, interest has grown in invertebrate defense systems. The immunological response is regulated by cytokines; however, while their role in mammals is well understood, little is known of their function in insects. A suitable target for studies into insect immunology is *Galleria mellonella* (Lepidoptera), the wax moth: a common host for human fungal and bacterial pathogens. *G. mellonella* is also a perfect subject for studies into the presence of cytokine-like proteins.

**Specific objectives:**

The main goal of present research was detection in insect immunocompetent cells the 18 mammalian cytokines (IL-1α, IL-1β, IL-2, IL-3, IL-6, IL-7, IL-8, IL-12, IL-13, IL-15, IL-17, IL-19, IFN-γ, TNF-α, TNF-β, GM-CSF, M-CSF, G-CSF), which play important role in immunological response and indication how their level change after fungal infection.

**Methodology:**

The changes of cytokine-like proteins level were detected in hemocytes taken from G*. mellonella* larvae infected with entomopathogenic fungus, *C. coronatus*. The presence of cytokine-proteins was confirmed with using fluorescence microscopy (in cultured hemocytes) and flow cytometry (in freshly collected hemolymph). The ELISA test was used to detect changes in concentration of examined cytokine-like proteins.

**Results:**

Our findings indicated the presence of eighteen cytokine-like molecules in *G. mellonella* hemocytes during infection with *C. coronatus*. The hemocytes taken from infected larvae demonstrated higher fluorescence intensity for six cytokine-like proteins (GM-CSF, M-CSF, IL-3, IL-15, IL-1β and IL-19) compared to untreated controls. ELISA test indicated significantly higher IL-3 and IL-15. M-CSF, IL-1α and IL-19 concentration in the hemolymph after fungal infection, and significantly lower TNF-β and G-CSF.

**Conclusions:**

Our findings confirm that the selected cytokine-like molecules are present in insect hemocytes and that their concentrations change after fungal infection, which might suggest that they play a role in the anti-fungal immunological response.

## Graphical Abstract

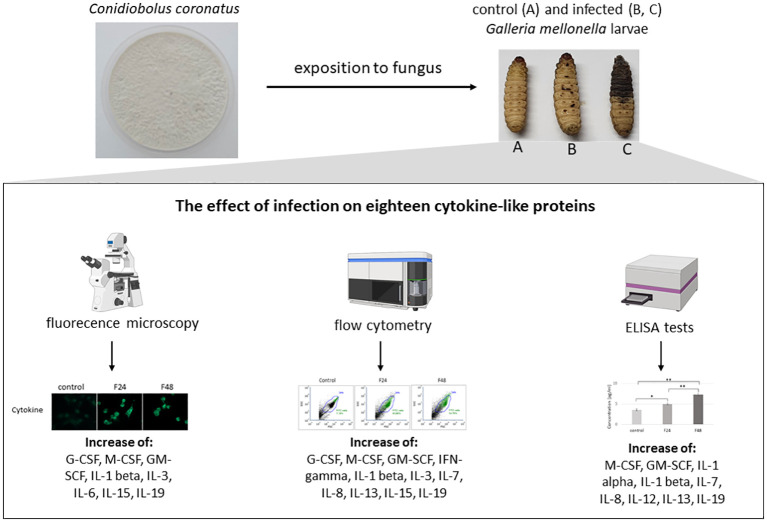

## 1 Introduction

Immunological studies have generally tended to favour the use of murine models, such as those based on rats. Nevertheless, due to the costly and labor-intensive nature of using such animals, and the necessity of maintaining large populations to acquire statistically significant data, there is an increasing demand for alternative models. One such possibility involves the use of those based on invertebrates (1). Comparative genome analyses in insects and other invertebrates have revealed a multitude of homologous genes to those found in humans, which encode proteins responsible for pathogen recognition or signal transduction. Hence, models based on insects such as *Drosophila melanogaster, Blattella germanica, Galleria mellonella, Culex quinquefasciatus* and *Bombyx mori* are becoming increasingly popular in studies on the virulence of microorganisms and host immunity (2).

Currently, the *G. mellonella* model (also called the wax worm or moth) is increasing in popularity in biological research. Several attributes of the larvae confer advantages as models: they are cost-effective to rear in abundance, straightforward to utilize, and require no specialized laboratory equipment for maintenance (3). The considerable size of the final instar larvae (12-20 mm) simplifies manipulation and enhances the ease of collecting tissue/hemolymph samples for analysis. Furthermore, administering test substances to the larvae is straightforward via food, topical application, or injection. The short life cycle of *G. mellonella*, approximately seven to eight weeks, makes them ideal for large-scale studies, especially since female wax moths can deposit around 1500 eggs in a single reproductive cycle. The temperature range at which these insects can be cultivated is crucial; unlike many other alternative invertebrate models, they thrive in a broad temperature range (18 - 37°C), with the length of the cycle influenced by temperature fluctuations (4). This characteristic is particularly valuable in immunological research as it enables mapping of temperature variations within mammalian bodies.

*G. mellonella* serves as a model host for human pathogens such as *Bacillus cereus* (5)*, Candida albicans* (6, 7), *Listeria monocytogenes* (8), and *Staphylococcus aureus* (9). Additionally, this insect is a popular model in research on entomopathogenic fungi activity (10) like *Conidiobolus coronatus* (Entomophthorales), a soil fungus pathogenic to insects and occasionally humans. In immunocompromised patients, particularly in tropical climates, *C. coronatus* can cause chronic infections, known as rhinofacial mycosis, characterized by invasion of adjacent skin and subcutaneous tissue in the face and nose, leading to deformity (11). *C. coronatus* as an entomopathogen selectively attacks various species of insects (12). Previous research on four medically significant fly species (*Calliphora vicina, Calliphora vomitoria, Lucilia sericata* (all Diptera: Calliphoridae), and *Musca domestica* (Diptera: Muscidae)) has revealed that while pupae exhibit resistance to *C. coronatus* infection, the adult flies are susceptible (13). The larvae of *C. vicina* have a thick cuticle that serves as a highly effective barrier against *C. coronatus* and exhibits poor degradation by proteases *in vitro*. The protective function of the cuticle is reinforced by the hemolymph, which has demonstrated effective antiproteolytic capabilities. However, the immune response of *C. vicina* is generally weak, marked by hemocytes with low phagocytic and encapsulating activity, an inefficient polyphenol oxidase (PO) system, and hemolymph with low lysozyme activity (14). On the contrary, despite possessing both humoral and cellular components in their immune systems, *G. mellonella* larvae are susceptible to *C. coronatus* infection due to their comparatively thin and readily degradable cuticle (15). Understanding the immune system’s response during infection is crucial for preventing and managing its effects.

The immunological system of *G. mellonella* larvae exhibits significant structural and functional resemblance to the innate immune response of mammals. Just as mammalian skin serves as a barrier to pathogens, the insect cuticle plays a similar role. Furthermore, the hemolymph of insects shares some similarities with mammalian blood, as both contain immunocompetent cells (16). While literature data did not confirm the presence of acquired immunity seen in mammals, which is characterized by the production of specific antibodies, they possess the capability to synthesize and secrete a range of antimicrobial peptides (AMPs) into the hemolymph (17). The humoral immune response can manifest through processes such as melanization, clotting, as well as production of reactive oxygen species (ROS) (18).

In *G. mellonella*, the cellular immune system rely on phagocytosis, nodulation, and encapsulation reactions and based on the presence of five distinct types of hemocytes, each contributing uniquely to the immune response. Plasmatocytes and granulocytes, the predominant cells, are known for their active phagocytic role. Conversely, oenocytoids, spherulocytes, and prohemocytes, though less explored, seem to have minor involvement in immune responses (19, 20).

While the factors governing immune responses in mammals are relatively well studied, there is little literature data describing these processes in insect. Nonetheless, it is widely described that their humoral immune responses mostly rely on antimicrobial peptides (AMPs) releasing from the fat body, which is mediated through pathways such as Toll, IMD (immune deficiency) and JAK-STAT (Janus kinase-signal transducer and activator of transcription). Toll signaling pathway is induced by Gram-positive bacteria and fungi, while Gram-negative bacteria mostly activate the IMD pathway (21).

The first stage of defence mechanisms in mammals are identification of molecular patterns detected in pathogens; in this phase the most important role plays the toll-like receptors (TLRs) present in dendritic cells, macrophages, and granulocytes, produced in hematopoietic stem cells.

Upon binding to a pathogen ligand, TLRs trigger a cascade of signaling pathways which are conserved from insects to plants and humans. They ultimately activate NF-κB (Nuclear Factor kappa B) which, in mammalian cells, leads to the induction of cytokine genes and the establishment of innate immunity (22). Within dendritic cells, TLR signals trigger the production of type I interferons (IFN α/β), which subsequently establish an antiviral state within host cells. Moreover, TLR signals elicit the release of both pro-inflammatory cytokines like IFNs, IL-1, TNF-α, and IL-12, and anti-inflammatory cytokines such as IL-10 and IL-6. Among these, IL-12 and IL-10 serve as connectors bridging early innate responses to specific immune responses (23).

The IMD pathway is very similar to the mammalian TNF-α pathway, a key regulator of vertebrate immunity and metabolism. Two well-described/examinated cellular reactions to TNF-α production include the initiation of apoptosis and the stimulation of transcription processes promoting cell survival. TNF-α-induced apoptosis is characterized by the activation of caspase cascades, leading to cell death through the cleavage of specific cellular substrates. Furthermore, TNF-α triggers the activation of two transcription factors: NF-κB and activating protein 1 (AP-1). Among these, NF-κB plays a crucial role in preventing apoptosis and ensuring cell survival by stimulating the expression of genes involved in inflammation, cell growth, and signal regulation (24).

The JAK-STAT pathway is one of the best understood signal transduction cascades. It is known to be universal and essential to cytokine receptor signaling. Almost 50 cytokine receptors realize their signals through combinations of four JAK and seven STAT family members, suggesting commonality across the JAK-STAT signaling system. Activation of this pathway induces cell proliferation, differentiation, migration, and apoptosis (25, 26).

The three main pathways for regulating cytokine activity in the insect immune system (Toll, IMD, JAK-STAT) have analogous equivalents in mammals. Therefore, we hypothesize that cytokine-like proteins may also be present in insects. The main goal of this study is to confirm the presence of eighteen cytokine-like molecules in *G. mellonella* hemocytes following *C. coronatus* infection.

## 2 Materials and methods

### 2.1 Culture of Galleria mellonella

A colony of *Galleria mellonella* wax moths (Lepidoptera: Pyralidae) was maintained in chambers with controlled temperature and humidity (30°C, 70% r.h.) without photoperiod (in constant darkness) and fed an artificial diet (ingredients: wheat flour, wheat bran, corn flour, skimmed milk powder, honey, glycerine) (27). Fully mature larvae were collected prior to pupation, subjected to surface sterilization and homogenization, and then utilized as an adjunct in fungal cultures. Five-day-old last instar larvae were employed to investigate the impact of fungal infection on the detection and concentration of various cytokine-like proteins, including Interleukin (IL)-1α, IL-1β, IL-2, IL-3, IL-6, IL-7, IL-8, IL-12, IL-13, IL-15, IL-17, IL-19, Interferon (IFN)-γ, Tumor necrosis factor (TNF)-α, TNF-β, granulocyte-macrophage colony-stimulating factor (GM-CSF), macrophage colony-stimulating factor (M-CSF), and granulocyte colony-stimulating factor (G-CSF), in both hemolymph and hemocytes.

### 2.2 Pure colony isolation and culture of *Conidiobolus coronatus*

*Conidiobolus coronatus* (isolate number 3491), initially isolated from *Dendrolaelaps* spp., was sourced from the collection of Prof. Bałazy at the Polish Academy of Sciences, Research Center for Agricultural and Forest Environment in Poznań. It was cultivated in 90 mm Petri dishes at 20°C in photoperiod (12-hour light:dark) for sporulation induction on Sabouraud agar medium (1% (w:v) peptone, 4% (w:v) glucose and 1.8% (w:v) agar with a final pH of 5.6) (SAM). Additionally, to increase the sporulation and virulence of the *C. coronatus* cultures, medium was supplemented with homogenized *G. mellonella* larvae at a final concentration of 10% wet weight.

### 2.3 Infection of insects with *C*. *coronatus*

*G*. *mellonella* larvae (five-day-old last instar) were exposed for 24 hours to even-day-old fully-grown and sporulating *C*. *coronatus* colonies. Twenty individuals were maintained in each Petri dish. A control group was formed of larvae exposed for 24 hours to sterile Sabouraud agar medium (Merck). After exposure, the insects were transferred to new, clean Petri dishes with appropriate food (an artificial diet (27)) and kept at 20°C for one day. Following this 24-hour exposure to the fungus, one group of insects was collected immediately for examination (F24 group) while the rest were left for another 24 hours before collection (F48 group).

### 2.4 Larval hemolymph collection

Hemolymph from both control and infected larvae (F24 and F48) of *G. mellonella* was collected. Due to the high percent of dead insect (68 ± 4.5% in F24 and 87 ± 5.2% in F48), the hemolymph samples were obtained from both surviving and dying individuals. In order to sterilize the surface of the larva and thus to reduce the contamination of hemolymph samples, the insects were first washed with 70% (v/v) ethanol and then briefly with distilled water. Hemolymph was collected from the larvae through an incision made in the last proleg. Depending on the planned research method, hemolymph was collected in different ways.

To culture the hemocytes, 100 μl of hemolymph taken from ten larvae was mixed with 500 μl of supplemented Grace’s Insect Medium (GIM; Invitrogen) containing antibiotics (gentamicin,10mg/ml; and amphotericin B (250μg/ml; both from Gibco), and phenylthiourea (PTU; 0.1mM; Sigma-Aldrich). Next samples were put in a six-channel μ-Slide IV 0.4 (IBIDI), 100μl of sample to each channel and incubated at 27°C for 24 hours.

For flow cytometric analysis, 100 μl of hemolymph taken from thirty larvae were mixed with 100 μl of supplemented GIM containing anticoagulant (10mM EDTA and 30mM sodium citrate).

For enzyme-linked immunosorbent assay (ELISA), 200 μl of hemolymph taken from 35 larvae was mixed with 100 μl of supplemented GIM medium. The hemocytes was firstly lysed during the sonication process (20 kHz, 3 min). and next centrifuged at 10000 x g for 10 min. The supernatants were put to new 1.5ml plastic tubes and stored at -20°C for further analysis.

### 2.5 Immunolocalization of cytokine-like proteins in *G. mellonella* hemocytes

The cytokine-like proteins IL-1α, IL-1β, IL-2, IL-3, IL-6, IL-7, IL-8, IL-12, IL-13, IL-15, IL-17, IL-19, IFN-γ, TNF-α, TNF-β, GM-CSF, M-CSF, G-CSF were subjected to immunolocalization of using fluorescence microscopy and flow cytometry. The same primary antibodies were used for both research methods: anti-IL-1α, anti-IL-1β, anti-IL-2, anti-IL-3, anti-IL-6, anti-IL-7, anti-IL-8, anti-IL-12, anti-IL-13, anti-IL-15, anti-IL-17, anti-IL-19 polyclonal antibodies were purchased from BioVision; anti-IFN-γ, anti-TNF-α, anti-TNF-β, anti-GM-CSF, anti-M-CSF, anti-G-CSF polyclonal antibodies were purchased from Invitrogen (a part of Thermo Fisher Scientific). Goat anti-Rabbit IgG (H+L), DyLight 488 (Invitrogen) or Goat anti-Mouse IgG (H+L), DyLight 488 (Invitrogen) was used as the secondary antibody depending on the primary antibody host.

### 2.6 Immunolocalization by fluorescence microscopy

Fluorescence microscopy was used to immunolocalize selected cytokine-like proteins in all hemocyte cultures (taken from controls, F24, and F48 insects). Firstly hemocytes were fixed (4% paraformaldehyde; Sigma-Aldrich; PFA) and permeabilized (0.1% Triton X-100;Sigma-Aldrich; both suspended in PBS). Next, the cells were incubated in 4% BSA-PBS for one hour to prevent a non-specific antibody binding. Primary antibodies listed above were then applied to hemocyte and incubate overnight at 4°C (1:60 suspended in PBS). After that, the cells were incubated for two hours at room temperature with secondary antibodies.

Concentrations of secondary antibody were 2μg/ml. ActinRed 555 ReadyProbes Reagent (Invitrogen) were used to label the actin fibers. The cell nuclei were stained with Hoechst (Enzo Life Sciences). Fluorescence signals were analyzed by fluorescent microscopy using an Axio Vert.A1 fluorescence microscope (Zeiss) with Axio Cam ICc 5 (Zeiss) and ZEN 3.2 lite software with Modul Image Analysis (Zeiss). Each test was performed in three independent replicates.

### 2.7 Flow cytometry analysis

Flow cytometry analysis was performed according to a protocol previously developed by our team for the determination of cytokine-like proteins in insect hemocytes (28). The hemolymph taken from both untreated and fungus-treated larvae was centrifuged (400xg, 10 min) and washed with PBS. The cells were then fixed in 4% paraformaldehyde (Sigma-Aldrich; PFA) in phosphate-buffered saline (PBS) and permeabilized in 0.1% Triton X-100 (Sigma-Aldrich) in PBS. After that, the cells were incubated with primary antibodies (diluted 1:100) overnight at 4°C, using the same antibodies as previously described. After three washes in PBS, the cells were further incubated for two hours at room temperature with the secondary antibody.

The readings were acquired on an CyFlow Cube 8 (Sysmex) and analyzed with FCS Express 6 (DeNovo Software). For each experimental condition, 100μl of each sample was scrutinized. Data was acquired using a 488 nm laser which detected each cytokine on the FL-1 channel. Each measurement was performed in three independent replicates. Results were shown as dot plots comparing forward scatter (FSC) with side scatter (SSC).

### 2.8 Cytokine-like proteins quantification by ELISA

Quantitative cytokine-like proteins analysis was carried out using ELISA tests all from Wuhan Fine Biotech Co., Ltd. The following commercial ELISA kits were used: Human IL-1α, Human IL-1β, Human IL-2, Human IL-3, Human IL-6, Human IL-7, Human IL-8, Human IL-12, Human IL-13, Human IL-15, Human IL-17, Human IL-19, Human IFN-γ, Human TNF-α, Human TNF-β, Human GM-CSF, Human M-CSF and Human G-CSF. Each test was performed in three independent replicates according to the manufacturer’s instructions.

### 2.9 BLASTP analysis

For preliminary proteomic analysis, the human amino acid sequences of the 18 studied cytokines (IL-1α, IL-1β, IL-2, IL-3, IL-6, IL-7, IL-8, IL-12, IL-13, IL-15, IL-17, IL-19, IFN-γ, TNF-α, TNF-β, GM-CSF, M-CSF, G-CSF) were compared with the *G. mellonella* proteomic database. Sequences of human cytokines acquired from UniProt [https://doi.org/10.1093/nar/gkac1052] where used as queries in blastp searches (BLASTP 2.12.0+) against UniProtKB reference genomes + Swiss-Prot databases with results restricted to *G. mellonella* (taxon ID 7137). Blastp searches were executed *via* the UniProt website (https://www.uniprot.org/blast) with default settings: Matrix: BLOSUM62; Gap Penalties: Existence: 11, Extension: 1; Neighboring words threshold: 11; Window for multiple hits: 40.

### 2.10 Statistics

Statistical analysis was conducted utilizing STATISTICA 6.1 software (StatSoft Polska). The one-way ANOVA was employed to assess statistical relationships, followed by Tukey’s test for *post hoc* analysis. Normality was examined using the Kolmogorov–Smirnov (K–S) test. The concentrations of cytokine-like proteins in hemolymph, measured by ELISA, were analyzed using Pearson’s correlation test, using OriginPro (OriginLab) software. In all cases, a p-value below 0.05 was considered statistically significant.

## 3 Results

The effects of *C. coronatus* infection on the amounts of eighteen cytokine-like proteins (IL-1α, IL-1β, IL-2, IL-3, IL-6, IL-7, IL-8, IL-12, IL-13, IL-15, IL-17, IL-19, IFN-γ, TNF-α, TNF-β, GM-CSF, M-CSF, G-CSF) in hemocytes were examined in three groups of *G. mellonella* larvae. In the experimental phase of the research, fungus-treated insects were employed. The larvae were divided into two groups, both of which underwent a 24-hour incubation period with the fungus. First one, named F24, was larvae immediately taken do the experiments after exposition to fungus, the second one (F48) the fungus-infected larvae were incubated additionally 24h on sterile petri dish and then used in experiments. The control insects were incubated with sterile Sabouraud agar medium. Following collection, all samples were subjected to examination by fluorescence microscopy, flow cytometry and ELISA.

### 3.1 Immunolocalization of cytokine-like proteins in hemocytes

Two methods were used to immunolocalize the eighteen proteins in *G. mellonella* hemocytes: fluorescence microscopy and flow cytometry.

**Figure 1:**
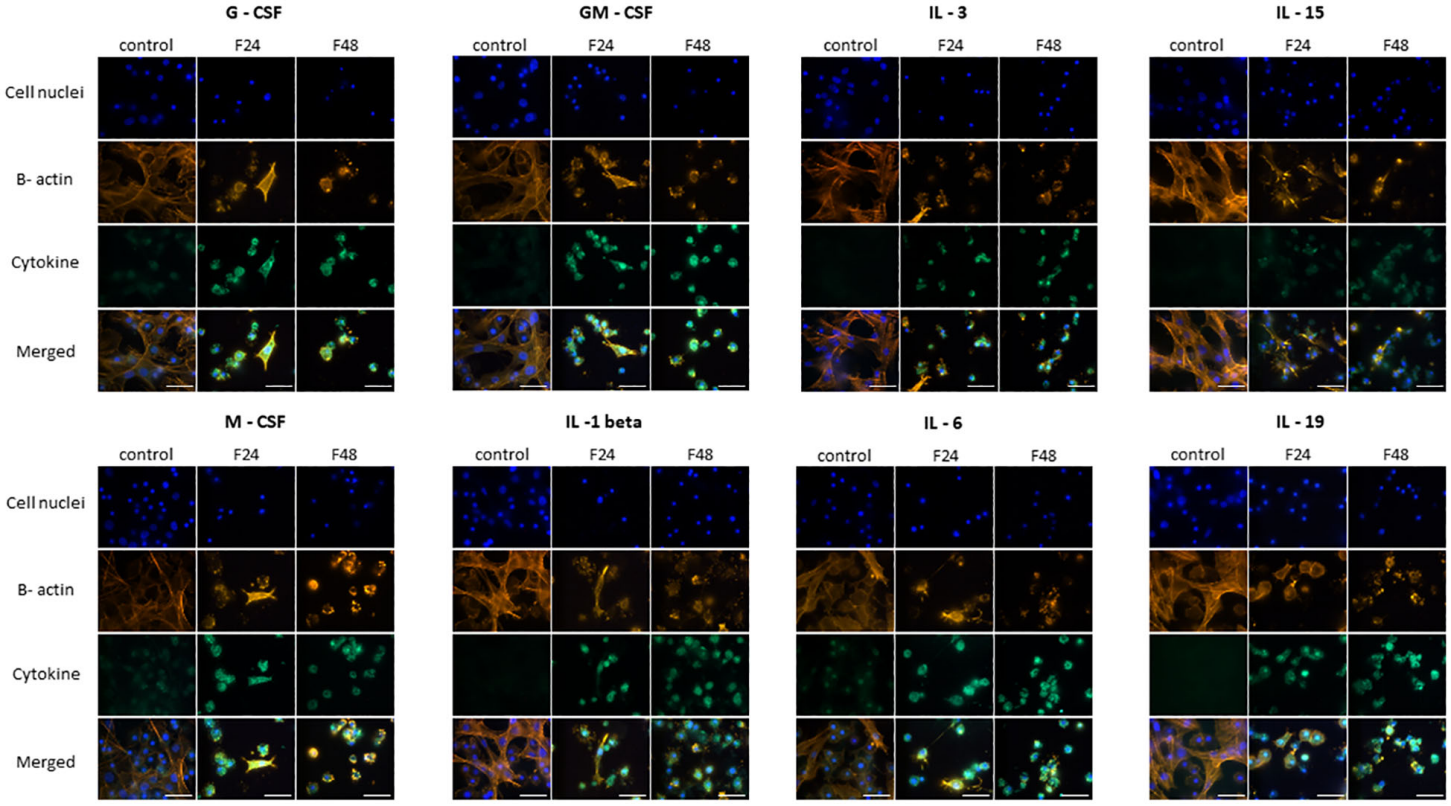
Immunofluorescence staining of G-CSF-, GM-CSF-, M-CSF-, IL-3-, IL-15-, IL-1 β-, IL-6-, IL-19- like proteins in *G. mellonella* hemocytes after *C. coronatus* infection. β-Actin (orange) was stained by ActinRed 555 ReadyProbes Reagent (Invitrogen). Cell nuclei (blue) were stained with Hoechst (Enzo Life Sciences). Cytokine-like proteins (green) were detected using the appropriate primary antibody, Goat anti-Mouse IgG (H+L), DyLight 488 (Invitrogen) as a secondary antibody for detecting G-CSF, and Goat anti-Rabbit IgG (H+L), DyLight 488 (Invitrogen) as secondary antibody for detecting the remaining cytokines. Control (negative control), non-infected, healthy larvae; F24, larvae sampled immediately after 24-h exposure to *C. coronatus* sporulating colonies; F48, larvae sampled 24 hours after 24-h exposure; scale bar 25 µm.

The fluorescence documentation depict labeled β-actin fibers, cell nuclei, immunolocalized cytokine-like proteins, and merged images. Microscopic research revealed two distinct hemocyte types: plasmatocytes and granulocytes. Other hemocyte subpopulations of *G. mellonella*, namely spherulocytes, oenocytoids, and prohemocytes, were non-adherent and consequently washed out during the fixation and staining processes. Of the proteins tested, eight in groups F24 and F48 (G-CSF, GM-CSF, M-CSF, IL-3, IL-15, IL-1 β, IL-6, IL-19) showed a significant increase in fluorescence intensity on the fluorescein isothiocyanate (FITC) channel compared to the control (**Figure 1**); this indicated an increase in their levels in hemocytes after fungal infection. However, no differences were found when comparing hemocytes collected from insects from groups F24 and F48.

**Figure 2:**
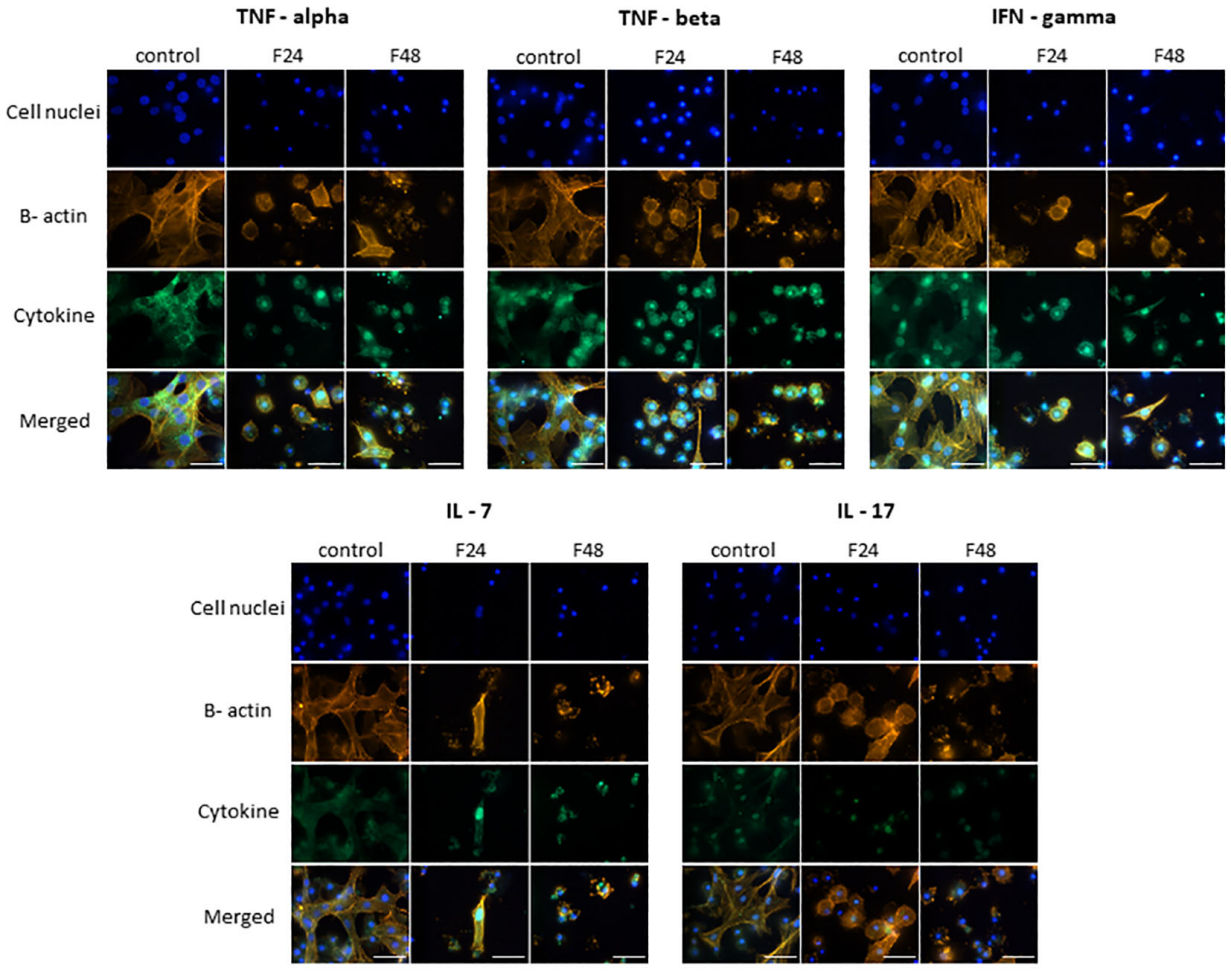
Immunofluorescence staining of TNF-α-, TNF-β-, IFN-gamma-, IL-7-, IL-17 – like proteins in *G. mellonella* hemocytes after *C. coronatus* infection. β-Actin (orange) was stained by ActinRed 555 ReadyProbes Reagent (Invitrogen). Cell nuclei (blue) were stained with Hoechst (Enzo Life Sciences). Cytokine-like proteins (green) were detected using the appropriate primary antibody, and Goat anti-Rabbit IgG (H+L), DyLight 488 (Invitrogen) as secondary antibody. Control (negative control), non-infected, healthy larvae; F24, larvae sampled immediately after 24-h exposure to *C. coronatus* sporulating colonies; F48, larvae sampled 24 hours after 24-h exposure; scale bar 25 µm.

TNF-α, TNF-β, IFN-gamma, IL-7 and IL-17 (**Figure 2**) demonstrated similar fluorescence intensities on the FITC channel in cytes collected from all three groups (control, F24 and F48). This confirms that their levels were not affected by fungal infection. In addition, it is worth emphasizing that IL-7 and IL-17 showed much lower green fluorescence compared to TNF-α, TNF-β and IFN-gamma.

**Figure 3:**
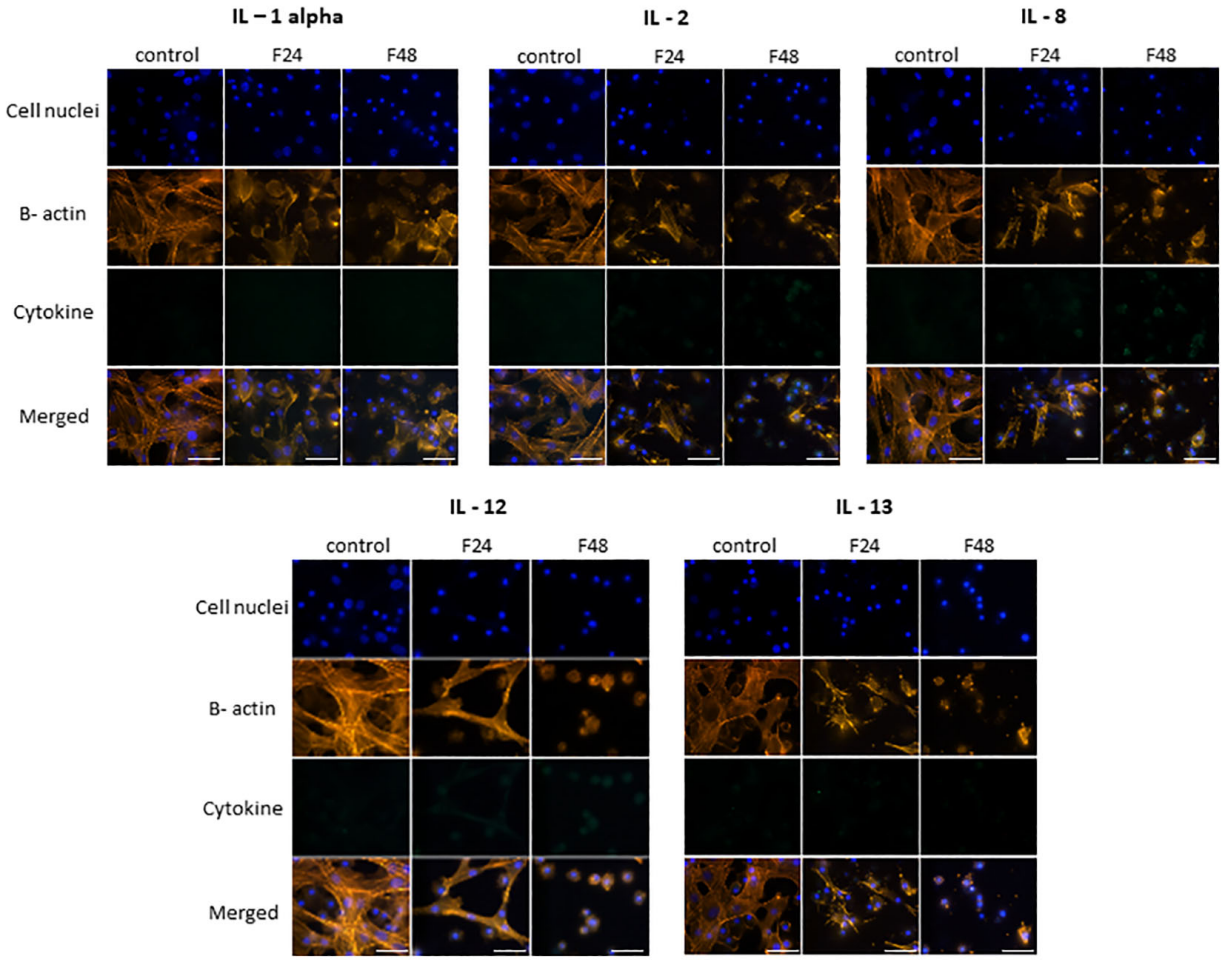
Immunofluorescence staining of IL-1 α-, IL-2-, IL-8-, IL-12-, IL-13 - like proteins in *G. mellonella* hemocytes after *C. coronatus* infection. β-Actin (orange) was stained by ActinRed 555 ReadyProbes Reagent (Invitrogen). Cell nuclei (blue) were stained with Hoechst (Enzo Life Sciences). Cytokine-like proteins (green) were detected using the appropriate primary antibody, Goat anti-Mouse IgG (H+L), DyLight 488 (Invitrogen) as a secondary antibody for the detection of IL-1 α and Goat anti-Rabbit IgG (H+L), DyLight 488 (Invitrogen) as secondary antibody for the detection of the remaining cytokines. Control (negative control), non-infected, healthy larvae; F24, larvae sampled immediately after 24-h exposure to *C. coronatus* sporulating colonies; F48, larvae sampled 24 hours after 24-h exposure; scale bar 25 µm.

**Figure 3** presents microscopic images of IL-1 α, IL-2, IL-8, IL-12 and IL-13 immunodetection. The lack of fluorescence on the FITC channel indicates the absence of these cytokine-like proteins in hemocytes from all cultures i.e. both healthy (control) and fungal-infected (F24 and F48) insects.

**Figure 4:**
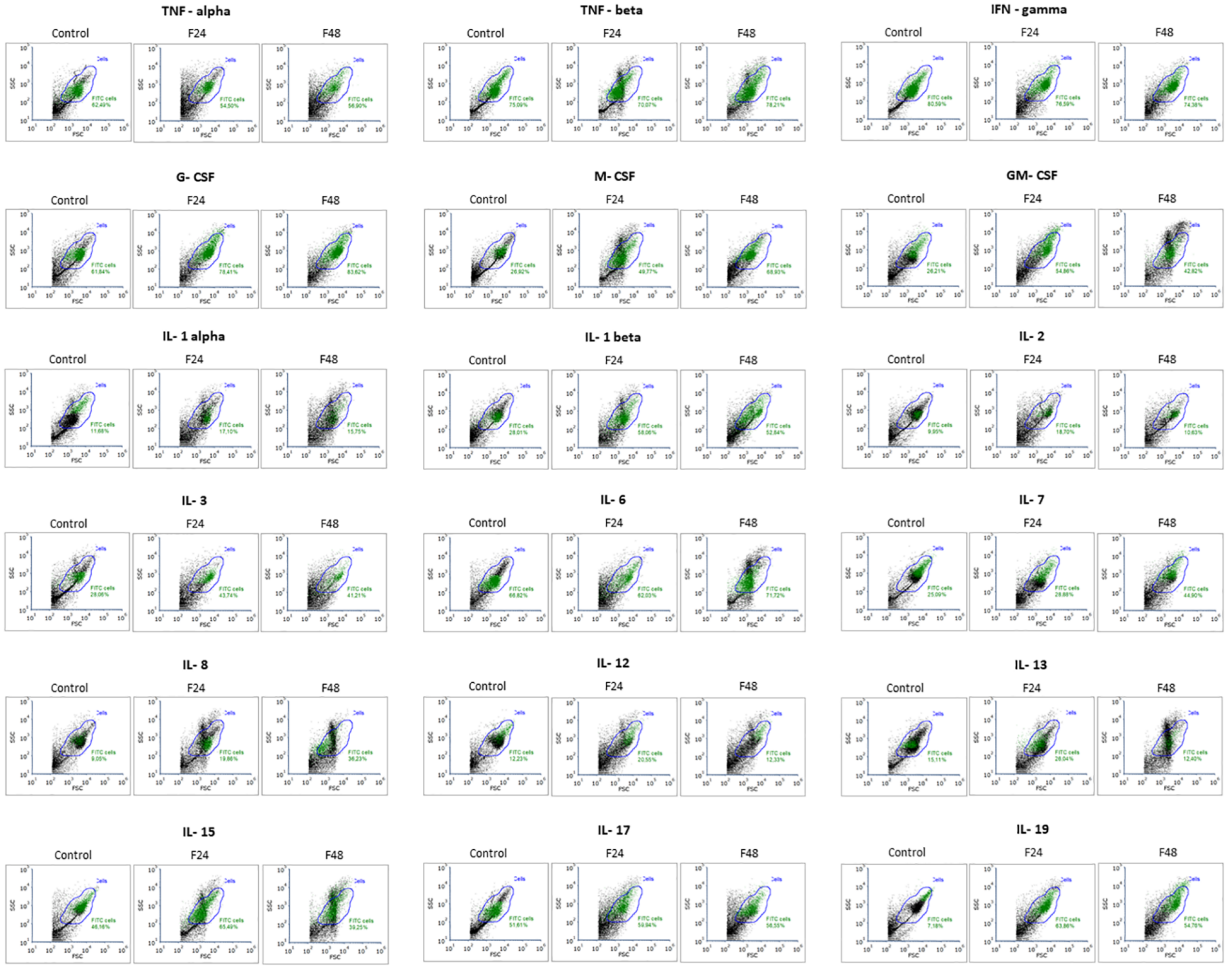
Flow cytometry analysis of cytokine like proteins IL-1α, IL-1β, IL-2, IL-3, IL-6, IL-7, IL-8, IL-12, IL-13, IL-15, IL-17, IL-19, IFN-γ, TNF-α, TNF-β, GM-CSF, M-CSF, G-CSF in *G. mellonella* hemocytes after *C. coronatus* infection. The results are shown as dot plots of FSC (forward scatter) versus SSC (side scatter). Gate ‘cells’ (blue)- all cells; gate ‘FITC cells’ (green)- cells that contain the tested protein. Control (negative control), non-infected, healthy larvae; F24, larvae sampled immediately after 24-h exposure to *C. coronatus* sporulating colonies; F48, larvae sampled 24 hours after 24-h exposure. One representative of three independent experiments is shown.

Flow cytometry data (**Figure 4**) are presented as dot plots of FSC (forward scatter) versus SSC (side scatter). All cells present in the sample are marked as gate ‘cells’ (blue), while cells that contain the tested protein were marked as ‘FITC cells’ (green; numerical data are provided in **Table S1**). Although all cell subpopulations were examined by this method, it was not possible to determine individual hemocyte subpopulations.

In the control group, the highest number of cells responding to antibody labeling was noted for IFN-gamma (82.92 ± 3.34%) and the lowest for IL-19 (7.65 ± 2.66%). Of the tested cytokines, G-CSF was the most prevalent among hemocytes taken from larvae from groups F24 (77.72 ± 1.83%) and F48 (82.91 ± 5.46%). In contrast, IL-2 was the least prevalent (F24 16.69 ± 5.21%; F48 10.16 ± 3.29%). In F24, significantly higher levels of M-CSF, GM-CSF, IL-1 β, IL-3, IL-8, IL-13, IL-15 and IL-19 positive cells were noted compared to control values; in addition, significantly higher levels of G-CSF, M-CSF, GM-CSF, IL-1 β, IL-7, IL-8 and IL-19 positive cells were found compared to both F48 and controls. The only statistically significant decrease between control and F48 was found for IFN-gamma. Detailed statistics (p-values for one-way ANOVA, with Tukey’s test) are presented in **Table S2**.

### 3.2 Quantitative measurement of cytokine-like protein concentrations in hemolymph

**Figure 5:**
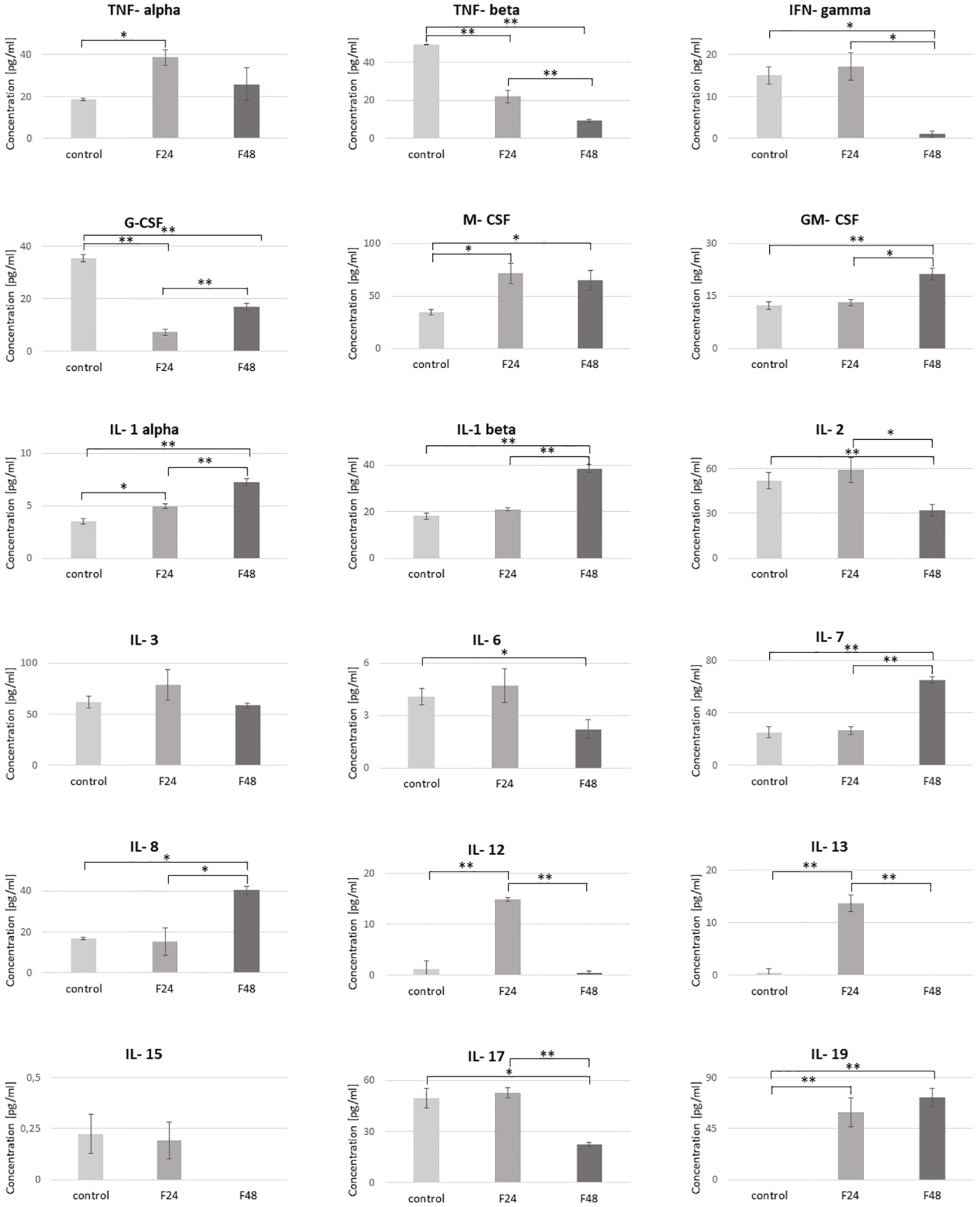
Concentration of cytokine-like proteins (IL-1α, IL-1β, IL-2, IL-3, IL-6, IL-7, IL-8, IL-12, IL-13, IL-15, IL-17, IL-19, IFN-γ, TNF-α, TNF-β, GM-CSF, M-CSF, G-CSF) in the hemolymph of *G. mellonella* larvae after infection with *C. coronatus*. The measurements were performed using ELISA tests. Data are presented as means and standard deviations. ∗p ≤ 0.05, ∗∗p < 0.001 (one-way ANOVA, with Tukey’s test, p ≤ 0.05). Control, non-infected, healthy larvae; F24, larvae sampled immediately after 24-h exposure to *C. coronatus* sporulating colonies; F48, larvae sampled 24 hours after 24-h exposure.

In healthy *G. mellonella* larvae (control) and those subjected to fungal infection (F24 and F48), the hemolymph concentrations of cytokine-like proteins (IL-1α, IL-1β, IL-2, IL-3, IL-6, IL-7, IL-8, IL-12, IL-13, IL-15, IL-17, IL-19, IFN-γ, TNF-α, TNF-β, GM-CSF, M-CSF, G-CSF) were determined using ELISA tests. The results are summarized in **Figure 5**.

The predominant cytokine-like protein in the control (61.92 ± 5.89 pg/ml) and F24 (78.99 ± 14.84 pg/ml) was IL-3, while IL-7 predominated in F48 (65.18 ± 2.36 pg/ml). Of the tested proteins, IL-19 was not detected in healthy controls, while IL-15 and IL-13 were not observed in F48.

No significant changes in concentration were found between healthy and infected insects for IL-3 and IL-15. However, M-CSF, IL-1 α and IL-19 were significantly higher in both infected groups (F24 and F48) compared with controls, while TNF-β and G-CSF were significantly lower. The IL-12 and IL-13 levels were also significantly higher, but only in the F24 group. Also significant differences between controls and F48 were found for IFN-gamma, GM-CSF, IL-1 β, IL-2, IL-6, IL-6, IL-7 and IL-17. The precise concentrations of the tested proteins are given in **Table S3**, with detailed statistics (p-values for one-way ANOVA, with Tukey’s test) in **Table S4**.

**Figure 6:**
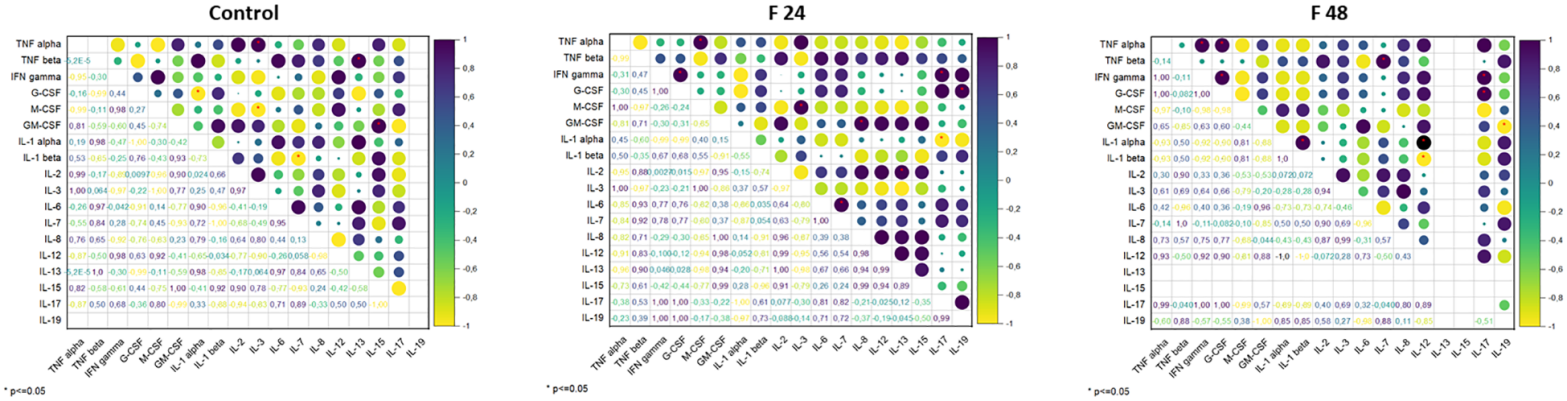
Pearson’s correlation coefficients (r) between the concentration of cytokine-like proteins (IL-1α, IL-1β, IL-2, IL-3, IL-6, IL-7, IL-8, IL-12, IL-13, IL-15, IL-17, IL-19, IFN-γ, TNF-α, TNF-β, GM-CSF, M-CSF, G-CSF). Positive correlations are indicated in shades of navy blue, whereas negative correlations are indicated in shades of yellow. Significant correlations, depending on the p-value, are indicated by red asterisks.

Pearson’s correlation coefficients were calculated between the levels of the tested proteins, in each individual group: control, F24 and F48 (**Figure 6**). In the controls, significant negative correlations were found between G-CSF *vs* IL-1 α, M-CSF *vs* IL-3, IL-1 β *vs* IL-7, and positive correlations between TNF- α *vs* IL-3, TNF-β *vs* IL-13, GM-CSF *vs* IL-15. In F24, a significant negative correlation was found for IL-1 α *vs* IL-17, and positive ones for IFN-gamma *vs* G-CSF, TNF- α *vs* M-CSF, IL-3 *vs* M-CSF, GM-CSF *vs* IL-8, IL-6 *vs* IL-7, IL-2 *vs* IL-13, IFN-gamma *vs* IL-17, G-CSF *vs* IL-19. In F48, significant negative correlations were found for IL-1 β *vs* IL-12 and GM-CSF *vs* IL-19, and positive ones for TNF-α *vs* IFN-gamma, TNF- α *vs* GM-CSF, IFN-gamma *vs* G-CSF, IL-1 α *vs* IL-1 β, TNF- β *vs* IL-7, IL-1 α *vs* IL-17, IFN-gamma *vs* IL-17 and G-CSF *vs* IL-17.

### 3.3 BLASTP analysis

The results of the preliminary proteomic analysis involving comparison of the amino acid sequence of 18 human cytokines (IL-1α, IL-1β, IL-2, IL-3, IL-6, IL-7, IL-8, IL-12, IL-13, IL-15, IL-17, IL-19, IFN-γ, TNF-α, TNF-β, GM-CSF, M-CSF, G-CSF) with *G. mellonella* proteomic databases using the BLASTP tool are presented in **Figure S1**, where the e-values below 0.01 considers homology between compared mammals and insect proteins. Uncharacterized proteins in *G. mellonella* proteomic databases showing homology (e-value <0.01) with human cytokines were detected for: IL-1α (one protein), IL-17, (one protein) and M-CSF (three proteins).

## 4 Discussion

In vertebrates, the responses to exogenous and endogenous insults, the tissue repair mechanisms and the restoration of tissue homeostasis are controlled by cytokines; as such they are regarded as the major regulators of immune processes (29, 30). Primarily these compounds are secreted by immunocytes, their function is believed to be mediated through interactions with particular cytokine receptors. Research on the functional similarities between the innate (non-adaptive) host defenses of vertebrates and invertebrates suggests that invertebrates possess soluble cytokine-like mediators that regulate inflammatory responses to infection or injury (31).

Although the topic of cytokines has been widely discussed in vertebrates, especially mammals (32), there is little information about cytokine-like proteins in invertebrates, and even less in insects (33). The present study examined the presence of 18 cytokine-like proteins (IL-1α, IL-1β, IL-2, IL-3, IL-6, IL-7, IL-8, IL-12, IL-13, IL-15, IL-17, IL-19, IFN-γ, TNF-α, TNF-β, GM-CSF, M-CSF, G-CSF) in the hemolymph of *G. mellonella* after infection with the entomopathogenic fungus *C. coronatus*. The research was carried out using three methods: fluorescence microscopy, flow cytometry and ELISA tests.

Thirteen of the tested proteins (G-CSF, GM-CSF, M-CSF, TNF-β, IFN-gamma, TNF-α, IL-1 β, IL-3, IL-6, IL-7, IL-15, IL-17, IL-19) were found in the hemolymph and hemocytes of *G. mellonella*; however, the results regarding the influence of infection differed according to the detection method. Fluorescence microscopy identified higher levels of G-CSF, GM-CSF, M-CSF, IL-3, IL-15, IL-1 β, IL-6, IL-19 in both F24 and F48 compared to controls. Flow cytometry analysis showed an increase in M-CSF, GM-CSF, IL-1 β, IL-8 and IL-19 for both post-infection samples compared to controls; in addition, ELISA assays indicated higher levels of M-CSF, GM-CSF and IL-1 α in the infected samples. These differences may result from the sample preparation method. All subpopulations of hemocytes can be detected by flow cytometry. However, fluorescence microscopy requires several washes during sample preparation, which may remove everything other than adherent subpopulations of hemocytes, i.e. plasmatocytes and granulocytes. In addition, ELISA tests are based on whole hemolymph, i.e. homogenized cells and plasma; however, this hemolymph was collected directly from the larvae and not subjected to *in vitro* cell culture.

Initial proteomic analysis conducted with the BLASTP tool revealed potential homology between three human cytokines (IL-1α, IL-17 and M-CSF) and uncharacterized proteins from *G. mellonella*, whose sequences were deposited in databases. For a more comprehensive assessment of the homology between human cytokines and insect cytokine-like proteins, a complete proteomic analysis is required. Our previous studies, in which analyzes were performed using 2D electrophoresis, Western Blot and LC-MS-MS/MS protein identification, confirmed 33% protein sequence coverage of *G. mellonella* IFN-γ- like protein to IFN-γ sequence from *Homo sapiens* (28). We are currently undertaking a proteomic analysis of larval hemolymph, utilizing FAIM (high-field asymmetric waveform ion mobility spectrometry) measurements and assessments through the Swissprot Eukaryota databases. We have obtained initial results into the identification of IL-17 and IL-19, which are undergoing further refinement for subsequent publication.

Previous immunocytochemical studies have confirmed the presence of cytokine-like proteins in various invertebrate species belonging to the Mollusca (34), Nematoda (35), Annelida (36), Tunicata (37) and Insecta (38). Of these, IL-1-, IL-6- and TNF-like molecules have been found to predominate in *inter alia* annelids, molluscs, echinoderms and protochordates (31, 39).

The IL-1-like cytokine was among the earliest-discover cytokine-like molecules in invertebrates, isolated from the coelomic fluid and coelomocytes of the starfish *Asterias forbesi* (40). In addition, molecules cross-reacting with antibodies against the chemokines IL-8, IL-1, IL-6 and IL-2, and against transforming growth factor (TGF)-β1, have been identified in molluscs (34, 41). Moreover, IL-2-like activity was observed in protochordates and echinoderms, specifically in deuterostome invertebrates possessing a hematopoietic organ and T-like cells (42). Finally, genes encoding TGF-β have been detected in the genome of cnidarians, such as the sea anemone *Aiptasia pallida*, and the cytokine was found to depress certain immune reactions, including nitric oxide production (43, 44).

Comparative studies indicated the presence of cytokine-like molecules in invertebrate and also similarities on genome level in both invertebrates and mammals, for example the research indicated the homology of mammalian messenger RNA (mRNA) to IL-1b mRNA isolated from cerebral ganglion of the protochordate *Stylea plicata* (45). In addition, PCR studies on *Manduca sexta* using primers based on shared vertebrate IL-1 protein sequences identified a product demonstrating 35% homology with sheep, rat, rabbit, cow, mouse and human IL-1α, IL-1β or IL-1-receptor antagonist (IL-1-ra) (46).

Few studies have reported the occurrence of these proteins in insects. However, a sequence homologous to an interferon consensus response element has been reported in the diptericin promoter of *Drosophila* (47), and a cytotoxic molecule (Gallysin 2) that may be an analogue of TNF has been isolated in the lepidopteran *G. mellonella* (48). Non-activated granular cells from *G. mellonella* or hemocytes from *Estigmene acraea* larvae have demonstrated strong positive reactions to anti-IL-1α and TNF-α polyclonal antibodies (pAb), while a less positive reaction was noted for *G. mellonella* plasmatocytes (49). In addition, TNF-like molecules have been observed in the plasmatocytes and granular cells of *Calliphora vomitoria* hemocytes (50). The presence of cytokine-like proteins in insects is also confirmed by our present findings; however, our study is the first to examine such a wide range of cytokines from different groups.

Similarly, very little is known of the effect of cytokine-like molecules on insects. Earthworm coelomocytes exhibited higher phagocytosis in response to recombinant human IL-12 and IFN-γ, whereas blue mussel hemocytes displayed increased stress and reduced phagocytosis when stimulated with TNF-α. Similarly, insect (*D. melanogaster*) cells demonstrated stimulation upon exposure to recombinant human IL-8, resulting in an increase in phagocytic cells (39). In addition, hemocytes of the fly *Calliphora vomitoria* demonstrated TNF-like molecule expression following encapsulation;the authors propose that his may serve as a chemoattractant (50). Finally, a defence complex consisting of IL-1-like molecule and phenol oxidase, the enzyme responsible for melanization, has been reported in the hemolymph of the insect *Manduca sexta* (51).

Infection by *C. coronatus* and the administration of its metabolites (harman and norharman) has been found to affect the insect immune system. Harman and norharman increase the phagocytic activity of hemocytes and the level of serotonin (5-HT) in *G. mellonella* hemolymph (15, 52). Our previous data identified the presence of certain heat shock proteins (HSPs), *viz.* HSP90, HSP70, HSP60 and HSP27, in *G. mellonella* hemocytes: HSP60 and HSP90 predominated in healthy insects, with HSP70 and HSP27 present in trace amounts, while HSP60 and HSP27 were elevated in the F24 and F48 groups, and HSP90 in F48 alone; the fungal infection had no effect on HSP70 levels (53). Both HSPs and serotonin are involved in regulating the functioning of the immune system. Heat shock proteins are activators of the innate immune system, and are capable of inducing pro-inflammatory cytokine production by mammalian macrophages (54). In contrast, 5-HT reduced IFN-γ and IL-17, and increased IL-10 production by T lymphocytes (55).

Insects mostly rely on their cuticular, humoral, and cellular defenses to combat fungal pathogens (56).

It is possible that the cytokine-like molecules present in insect hemolymph also inhibit fungal infection. In mammals, the role of cytokines in fungal infection is widely described: the cells of the host innate immune system recognize fungal organisms by their cell wall components, which act as pathogen‐associated molecular patterns (PAMPs). The PAMPs bind to, and are recognized by, pattern‐recognition receptors (PRRs), including Toll‐like receptors (TLRs), on the surface of host cells (57, 58).

The phagocytic activity directed against fungi takes place through oxidative and non‐oxidative mechanisms, and can be increased by opsonins and T‐cell‐derived cytokines (59). The production and activation of mature phagocytic cells from hematopoietic progenitor cells is stimulated by a group of glycoproteins known as hematopoietic growth factors (HGFs). Of the HGFs, the most relevant to antifungal host defenses are granulocyte colony‐stimulating factor (G‐CSF), granulocyte‐macrophage colony‐stimulating factor (GM‐CSF) and macrophage colony‐stimulating factor (M‐CSF) (60). Our present findings indicate that these cytokines are elevated in *G. mellonella* hemocytes after fungal infection, while our previous studies confirm that *C. coronatus* metabolites (harman and norharman) stimulate phagocytosis in these cells (52).

HGFs have been proven to provide defense against fatal fungal infections and bolster the effectiveness of antifungal medications in both animal models and clinical investigations. G-CSF, M-CSF, and GM-CSF have been employed in the management of invasive fungal infections stemming from Candida (61, 62) and Aspergillus (63–66), especially in patients undergoing chemotherapy or after stem cell transplantation (67–69). As such, the fact that these proteins also play a potential role in fungal infection in insects suggests supports their attractiveness as potential research models.

In mammals, two subpopulations of T helper cells are involved in the response to fungal infection: ‘protective’ type‐1 (Th1) and ‘non‐protective’ type‐2 (Th2). The type‐1 response involves the production of Th1 cytokines, such as IFN‐γ and IL‐2, IL‐12, and IL‐18; these stimulate macrophage activation, cytotoxic CD4+ T cell generation, opsonizing antibody production and delayed-type hypersensitivity. The type‐2 response entails the generation of Th2 cytokines, like IL‐4, IL‐5, and IL‐13, which trigger the production of non‐opsonizing antibodies and allergic reactions, while also suppressing the pronounced inflammatory response induced by Th1 cytokines. Several other cytokines contribute to the immune response against fungal pathogens, including IL‐1, IL‐6, IL‐8, IL‐10, IL‐15, TNF‐α, and transforming growth factor (TGF)‐β (70).

Our research with the *G. mellonella* insect model showed that *C. coronatus* infection did not appear to increase the levels of IFN-gamma-like protein and TNF‐α-like protein, as these proteins were produced by hemocytes from both healthy and infected insects. Nevertheless, as in mammals, it is possible that the IL-1-β, IL-6 and IL-15-like proteins may play an important role in fungal infection, as their levels increased in infected insects; however, a better understanding of the role of these proteins during infection is needed.

IL-1β signaling promotes monocyte, macrophage and neutrophil recruitment in mammals, as well as enhanced phagocytosis and killing, and increased production of reactive oxygen species/nitrogen oxide synthase (ROS/NOS) (71). IL-15 plays a role in innate immunity against fungal infections by boosting the antifungal activity of polymorphonuclear or monocyte cells (72); it also recently has been found to play a part in NK cell activation (73). IL-6 can stimulate the secretion of various chemokines, resulting in the recruitment of monocytes and/or macrophages and the resolution of inflammation (74).

## 5 Conclusions

Many similarities have been noted between the immune systems of mammals and insects, particularly regarding the functioning of immunocompetent cells, immune pathways and factors. This has been confirmed by our present findings, indicating that certain cytokine-like molecules are present in the insect hemocyte subpopulation and that their level correlates with the degree of fungal infection. These findings may also be valuable in contributing to further research concerning insect physiology, parasitology and immunology, as well as pest biocontrol.

## Data availability statement

The original contributions presented in the study are included in the article/**Supplementary Material**. Further inquiries can be directed to the corresponding author.

## Ethics statement

Ethical approval was not required for the study involving animals in accordance with the local legislation and institutional requirements because The research was conducted on invertebrates.

## Author contributions

AW: Conceptualization, Data curation, Formal analysis, Funding acquisition, Investigation, Methodology, Visualization, Writing – original draft. AK: Data curation, Formal analysis, Investigation, Writing – review & editing. JS: Investigation, Visualization, Writing – review & editing. MB: Methodology, Supervision, Writing – review & editing.

## References

[1] Huang X Liu Y Xi L Zeng K Mylonakis E. Galleria mellonella as a model invertebrate host for the study of muriform cells of dematiaceous fungi. Future Microbiol. (2018) 13:1021–8. doi: 10.2217/fmb-2018-0036 29927339

[2] Wilson-Sanders SE. Invertebrate models for biomedical research, testing, and education. ILAR J. (2011) 52:126–52. doi: 10.1093/ilar.52.2.126 21709307

[3] Binder U Maurer E Lass-Florl C. Galleria mellonella: An invertebrate model to study pathogenicity in correctly defined fungal species. Fungal Biol. (2016) 120:288–95. doi: 10.1016/j.funbio.2015.06.002 26781383

[4] Cook SM McArthur JD. Developing Galleria mellonella as a model host for human pathogens. Virulence. (2013) 4:350–3. doi: 10.4161/viru.25240 PMC3714126 23799664

[5] Nyamjav I Jang Y Park N Lee YE Lee S. Physicochemical and Structural Evidence that Bacillus cereus Isolated from the Gut of Waxworms (Galleria mellonella Larvae) Biodegrades Polypropylene Efficiently *In Vitro* . J Polym Environ. (2023) 10:1–14. doi: 10.21203/rs.3.rs-2536512/v1 PMC10171730 37361349

[6] de Souza PC Corrêa AEDN Gameiro JG de Oliveira Júnior AG Panagio LA Venancio EJ. Production of IgY against iron permease Ftr1 from Candida albicans and evaluation of its antifungal activity using Galleria mellonella as a model of systemic infection. Microb Pathog. (2023) 181:106166. doi: 10.1016/j.micpath.2023.106166 37290729

[7] Mannala GK Rupp M Walter N Scholz KJ Simon M Riool M. Galleria mellonella as an alternative in *vivo* model to study implant-associated fungal infections. J Orthop Res. (2023) 41:2547–59. doi: 10.1002/jor.25572 37080929

[8] Pan X Shen J Hong Y Wu Y Guo D Zhao L. Comparative analysis of growth, survival, and virulence characteristics of listeria monocytogenes isolated from imported meat. Microorganisms. (2024) 12. doi: 10.3390/microorganisms12020345 PMC10891628 38399749

[9] Alnezary FS Almutairi MS Alhifany AA Almangour TA. Assessing Galleria mellonella as a preliminary model for systemic Staphylococcus aureus infection: Evaluating the efficacy and impact of vancomycin and Nigella sativa oil on gut microbiota. Saudi Pharm J. (2023) 31:101824. doi: 10.1016/j.jsps.2023.101824 37965487 PMC10641552

[10] Desalermos A Fuchs BB Mylonakis E. Selecting an invertebrate model host for the study of fungal pathogenesis. PloS Pathog. (2012) 8:e1002451. doi: 10.1371/journal.ppat.1002451 22319439 PMC3271057

[11] Fischer N Ruef C Ebnother C Bachli EB. Rhinofacial Conidiobolus coronatus infection presenting with nasal enlargement. Infection. (2008) 36:594–6. doi: 10.1007/s15010-008-8056-5 18998052

[12] Bogus MI Scheller K. Extraction of an insecticidal protein fraction from the parasitic fungus Conidiobolus coronatus (Entomophthorales). Acta Parasitol. (2002) 44:66–72.

[13] Bogus MI Kedra E Bania J Szczepanik M Czygier M Jabłoński P. Cuticle hydrolysis in four medically important fly species by enzymes of the entomopathogenic fungus Conidiobolus coronatus. Med Vet Entomol. (2017) 31:23–35. doi: 10.1111/mve.12202 27770452

[14] Bogus MI Kedra E Bania J Szczepanik M Czygier M Jabłoński P. Different defense strategies of Dendrolimus pini, Galleria mellonella, and Calliphora vicina against fungal infection. J Insect Physiol. (2007) 53:909–22. doi: 10.1016/j.jinsphys.2007.02.016 17512001

[15] Wronska AK Boguś MI Włóka E Kazek M Kaczmarek A Zalewska K. Cuticular fatty acids of Galleria mellonella (Lepidoptera) inhibit fungal enzymatic activities of pathogenic Conidiobolus coronatus. PloS One. (2018) 13:e0192715. doi: 10.1371/journal.pone.0192715 29518079 PMC5843172

[16] Jiang H Vilcinskas A Kanost MR. Immunity in lepidopteran insects. Adv Exp Med Biol. (2010) 708:181–204. doi: 10.1007/978-1-4419-8059-5_10 21528699 PMC9284565

[17] Staczek S Cytrynska M Zdybicka-Barabas A. Unraveling the role of antimicrobial peptides in insects. Int J Mol Sci. (2023) 24. doi: 10.3390/ijms24065753 PMC10059942 36982826

[18] Sheehan G Garvey A Croke M Kavanagh K. Innate humoral immune defences in mammals and insects: The same, with differences? Virulence. (2018) 9:1625–39. doi: 10.1080/21505594.2018.1526531 PMC7000196 30257608

[19] Marmaras VJ Lampropoulou M. Regulators and signalling in insect haemocyte immunity. Cell Signal. (2009) 21:186–95. doi: 10.1016/j.cellsig.2008.08.014 18790716

[20] Browne N Heelan M Kavanagh K. An analysis of the structural and functional similarities of insect hemocytes and mammalian phagocytes. Virulence. (2013) 4:597–603. doi: 10.4161/viru.25906 23921374 PMC3906293

[21] Kingsolver MB Hardy RW. Making connections in insect innate immunity. Proc Natl Acad Sci U.S.A. (2012) 109:18639–40. doi: 10.1073/pnas.1216736109 PMC3503154 23100537

[22] Wardyn JD Ponsford AH Sanderson CM. Dissecting molecular cross-talk between Nrf2 and NF-kappaB response pathways. Biochem Soc Trans. (2015) 43:621–6. doi: 10.1042/BST20150014 PMC4613495 26551702

[23] Ozato K Tsujimura H Tamura T. Toll-like receptor signaling and regulation of cytokine gene expression in the immune system. Biotechniques. (2002) 70:66–68. doi: 10.2144/Oct0208 12395929

[24] Davoodi S Galenza A Panteluk A Deshpande R Ferguson M Grewal S. The immune deficiency pathway regulates metabolic homeostasis in drosophila. J Immunol. (2019) 202:2747–59. doi: 10.4049/jimmunol.1801632 30902902

[25] Murray PJ. The JAK-STAT signaling pathway: input and output integration. J Immunol. (2007) 178:2623–9. doi: 10.4049/jimmunol.178.5.2623 17312100

[26] Morris R Kershaw NJ Babon JJ. The molecular details of cytokine signaling via the JAK/STAT pathway. Protein Sci. (2018) 27:1984–2009. doi: 10.1002/pro.3519 30267440 PMC6237706

[27] Sehnal F. A critical study of the biome and biometry of the wax moth Galleria mellonella raised in varying conditions. Z für wissenschaftliche Zool. (1966) 174:53–82.

[28] Wronska AK Kaczmarek A Sobich J Grzelak S Bogus MI. Intracellular cytokine detection based on flow cytometry in hemocytes from Galleria mellonella larvae: A new protocol. PloS One. (2022) 17:e0274120. doi: 10.1371/journal.pone.0274120 36173940 PMC9521830

[29] Toomer KH Malek TR. Cytokine signaling in the development and homeostasis of regulatory T cells. Cold Spring Harb Perspect Biol. (2018) 10. doi: 10.1101/cshperspect.a028597 PMC5830895 28620098

[30] Legrand JMD Martino MM. Growth factor and cytokine delivery systems for wound healing. Cold Spring Harb Perspect Biol. (2022) 14. doi: 10.1101/cshperspect.a041234 PMC9341469 35667794

[31] Beschin A Bilej M Torreele E De Baetselier P. On the existence of cytokines in invertebrates. Cell Mol Life Sci. (2001) 58:801–14. doi: 10.1007/PL00000901 PMC11337351 11437239

[32] Liu C Chu D Kalantar-Zadeh K George J Young HA Liu G. Cytokines: from clinical significance to quantification. Adv Sci (Weinh). (2021) 8:e2004433. doi: 10.1002/advs.202004433 34114369 PMC8336501

[33] Gerardo NM Hoang KL Stoy KS. Evolution of animal immunity in the light of beneficial symbioses. Philos Trans R Soc Lond B Biol Sci. (2020) 375:20190601. doi: 10.1098/rstb.2019.0601 32772666 PMC7435162

[34] Qiao X Wang L Song L. The primitive interferon-like system and its antiviral function in molluscs. Dev Comp Immunol. (2021) 118:103997. doi: 10.1016/j.dci.2021.103997 33444647

[35] Chowanski S Walkowiak-Nowicka K Winkiel M Marciniak P Urbański A Pacholska-Bogalska J. Insulin-like peptides and cross-talk with other factors in the regulation of insect metabolism. Front Physiol. (2021) 12:701203. doi: 10.3389/fphys.2021.701203 34267679 PMC8276055

[36] Grimaldi A Banfi S Bianchi C Gabriella G Tettamanti G Noonan DM. The leech: a novel invertebrate model for studying muscle regeneration and diseases. Curr Pharm Des. (2010) 16:968–77. doi: 10.2174/138161210790883417 20041825

[37] Raftos D Nair S. Tunicate cytokine-like molecules and their involvement in host defense responses. Prog Mol Subcell Biol. (2004) 34:165–82. doi: 10.1007/978-3-642-18670-7_7 14979668

[38] Kaczmarek A Wronska AK Kazek M Bogus MI. Octanoic Acid-An Insecticidal Metabolite of Conidiobolus coronatus (Entomopthorales) That Affects Two Majors Antifungal Protection Systems in Galleria mellonella (Lepidoptera): Cuticular Lipids and Hemocytes. Int J Mol Sci. (2022) 23. doi: 10.3390/ijms23095204 PMC9101785 35563592

[39] Buchmann K. Evolution of innate immunity: clues from invertebrates via fish to mammals. Front Immunol. (2014) 5:459. doi: 10.3389/fimmu.2014.00459 25295041 PMC4172062

[40] Beck G Habicht GS. Isolation and characterization of a primitive interleukin-1-like protein from an invertebrate, Asterias forbesi. Proc Natl Acad Sci U.S.A. (1986) 83:7429–33. doi: 10.1073/pnas.83.19.7429 PMC386731 3489938

[41] Sun J Wang L Song L. The primitive complement system in molluscs. Dev Comp Immunol. (2023) 139:104565. doi: 10.1016/j.dci.2022.104565 36216083

[42] Kondo M Akasaka K. Current Status of Echinoderm Genome Analysis - What do we Know? Curr Genomics. (2012) 13:134–43. doi: 10.2174/138920212799860643 PMC3308324 23024605

[43] Seneca F Davtian D Boyer L Czerucka D. Gene expression kinetics of Exaiptasia pallida innate immune response to Vibrio parahaemolyticus infection. BMC Genomics. (2020) 21:768. doi: 10.1186/s12864-020-07140-6 33167855 PMC7654579

[44] Detournay O Schnitzler CE Poole A Weis VM. Regulation of cnidarian-dinoflagellate mutualisms: Evidence that activation of a host TGFbeta innate immune pathway promotes tolerance of the symbiont. Dev Comp Immunol. (2012) 38:525–37. doi: 10.1016/j.dci.2012.08.008 23010490

[45] Pestarino M De Anna E Masini MA Sturla M. Localization of interleukin-1 beta mRNA in the cerebral ganglion of the protochordate, Styela plicata. Neurosci Lett. (1997) 222:151–4. doi: 10.1016/s0304-3940(97)13362-6 9148237

[46] Beck G. Macrokines:invertebrate cytokine-like molecules? Front Biosci. (1998) 3:d559–569. doi: 10.2741/a303 9628939

[47] Georgel P Kappler C Langley E Gross I Nicolas E Reichhart JM. Drosophila immunity. A sequence homologous to mammalian interferon consensus response element enhances the activity of the diptericin promoter. Nucleic Acids Res. (1995) 23:1140–5. doi: 10.1093/nar/23.7.1140 PMC306822 7537872

[48] Beresford PJ Basinski-Gray JM Chiu JK Chadwick JS Aston WP. Characterization of hemolytic and cytotoxic Gallysins: a relationship with arylphorins. Dev Comp Immunol. (1997) 21:253–66. doi: 10.1016/s0145-305x(97)00011-6 9258607

[49] Wittwer D Franchini A Ottaviani E Wiesner A. Presence of IL-1- and TNF-like molecules in Galleria mellonella (Lepidoptera) haemocytes and in an insect cell line Fromestigmene acraea (Lepidoptera). Cytokine. (1999) 11:637–42. doi: 10.1006/cyto.1998.0481 10479399

[50] Franchini A Miyan JA Ottaviani E. Induction of ACTH- and TNF-alpha-like molecules in the hemocytes of Calliphora vomitoria (Insecta, Diptera). Tissue Cell. (1996) 28:587–92. doi: 10.1016/s0040-8166(96)80061-9 8858884

[51] Beck G Cardinale S Wang L Reiner M Sugumaran M. Characterization of a defense complex consisting of interleukin 1 and phenol oxidase from the hemolymph of the tobacco hornworm, Manduca sexta. J Biol Chem. (1996) 271:11035–8. doi: 10.1074/jbc.271.19.11035 8626641

[52] Wronska AK Bogus MI. Harman and norharman, metabolites of the entomopathogenic fungus Conidiobolus coronatus (Entomophthorales), affect the serotonin levels and phagocytic activity of hemocytes, insect immunocompetent cells, in Galleria mellonella (Lepidoptera). Cell Biosci. (2019) 9:29. doi: 10.1186/s13578-019-0291-1 30962871 PMC6434831

[53] Wronska AK Bogus MI. Heat shock proteins (HSP 90, 70, 60, and 27) in Galleria mellonella (Lepidoptera) hemolymph are affected by infection with Conidiobolus coronatus (Entomophthorales). PloS One. (2020) 15:e0228556. doi: 10.1371/journal.pone.0228556 32027696 PMC7004346

[54] Gao B Tsan MF. Induction of cytokines by heat shock proteins and endotoxin in murine macrophages. Biochem Biophys Res Commun. (2004) 317:1149–54. doi: 10.1016/j.bbrc.2004.03.160 15094389

[55] Sacramento PM Monteiro C Dias ASO Kasahara TM Ferreira TB Hygino J. Serotonin decreases the production of Th1/Th17 cytokines and elevates the frequency of regulatory CD4(+) T-cell subsets in multiple sclerosis patients. Eur J Immunol. (2018) 48:1376–88. doi: 10.1002/eji.201847525 29719048

[56] Qu S Wang S. Interaction of entomopathogenic fungi with the host immune system. Dev Comp Immunol. (2018) 83:96–103. doi: 10.1016/j.dci.2018.01.010 29355579

[57] Behzadi P Garcia-Perdomo HA Karpinski TM. Toll-like receptors: general molecular and structural biology. J Immunol Res. (2021) 2021:9914854. doi: 10.1155/2021/9914854 34195298 PMC8181103

[58] Taghavi M Khosravi A Mortaz E Nikaein D Athari SS. Role of pathogen-associated molecular patterns (PAMPS) in immune responses to fungal infections. Eur J Pharmacol. (2017) 808:8–13. doi: 10.1016/j.ejphar.2016.11.013 27851904

[59] Huffnagle GB Deepe GS. Innate and adaptive determinants of host susceptibility to medically important fungi. Curr Opin Microbiol. (2003) 6:344–50. doi: 10.1016/S1369-5274(03)00089-4 12941402

[60] Mansour MK Levitz SM. Interactions of fungi with phagocytes. Curr Opin Microbiol. (2002) 5:359–65. doi: 10.1016/S1369-5274(02)00342-9 12160853

[61] Kullberg BJ Netea MG Vonk AG van der Meer JW. Modulation of neutrophil function in host defense against disseminated Candida albicans infection in mice. FEMS Immunol Med Microbiol. (1999) 26:299–307. doi: 10.1111/fim.1999.26.issue-3-4 10575142

[62] Kuhara T Uchida K Yamaguchi H. Therapeutic efficacy of human macrophage colony-stimulating factor, used alone and in combination with antifungal agents, in mice with systemic Candida albicans infection. Antimicrob Agents Chemother. (2000) 44:19–23. doi: 10.1128/AAC.44.1.19-23.2000 10602717 PMC89622

[63] Gonzalez CE Lyman CA Lee S Del Guercio C Roilides E Bacher J. Recombinant human macrophage colony-stimulating factor augments pulmonary host defences against Aspergillus fumigatus. Cytokine. (2001) 15:87–95. doi: 10.1006/cyto.2001.0889 11500084

[64] Brummer E Maqbool A Stevens DA. *In vivo* GM-CSF prevents dexamethasone suppression of killing of Aspergillus fumigatus conidia by bronchoalveolar macrophages. J Leukoc Biol. (2001) 70:868–72. doi: 10.1189/jlb.70.6.868 11739548

[65] Quezada G Koshkina NV Zweidler-McKay P Zhou Z Kontoyiannis DP Kleinerman ES. Intranasal granulocyte-macrophage colony-stimulating factor reduces the Aspergillus burden in an immunosuppressed murine model of pulmonary aspergillosis. Antimicrob Agents Chemother. (2008) 52:716–8. doi: 10.1128/AAC.00760-07 PMC2224773 17984233

[66] Sionov E Mendlovic S Segal E. Experimental systemic murine aspergillosis: treatment with polyene and caspofungin combination and G-CSF. J Antimicrob Chemother. (2005) 56:594–7. doi: 10.1093/jac/dki252 16006446

[67] Giles FJ. Monocyte-macrophages, granulocyte-macrophage colony-stimulating factor, and prolonged survival among patients with acute myeloid leukemia and stem cell transplants. Clin Infect Dis. (1998) 26:1282–9. doi: 10.1086/516361 9636847

[68] Volpi I Perruccio K Tosti A Capanni M Ruggeri L Posati S. Postgrafting administration of granulocyte colony-stimulating factor impairs functional immune recovery in recipients of human leukocyte antigen haplotype-mismatched hematopoietic transplants. Blood. (2001) 97:2514–21. doi: 10.1182/blood.v97.8.2514 11290617

[69] Ringden O Labopin M Gorin NC Le Blanc K Rocha V Gluckman E. Treatment with granulocyte colony-stimulating factor after allogeneic bone marrow transplantation for acute leukemia increases the risk of graft-versus-host disease and death: a study from the Acute Leukemia Working Party of the European Group for Blood and Marrow Transplantation. J Clin Oncol. (2004) 22:416–23. doi: 10.1200/JCO.2004.06.102 14691124

[70] Romani L. Immunity to fungal infections. Nat Rev Immunol. (2011) 11:275–88. doi: 10.1038/nri2939 21394104

[71] Griffiths JS Camilli G Kotowicz NK Ho J Richardson JP Naglik JR. Role for IL-1 family cytokines in fungal infections. Front Microbiol. (2021) 12:633047. doi: 10.3389/fmicb.2021.633047 33643264 PMC7902786

[72] Winn RM Gil-Lamaignere C Roilides E Simitsopoulou M Lyman CA Maloukou A. Selective effects of interleukin (IL)-15 on antifungal activity and IL-8 release by polymorphonuclear leukocytes in response to hyphae of Aspergillus species. J Infect Dis. (2003) 188:585–90. doi: 10.1086/377099 12898448

[73] Tran P Ahmad R Xu J Ahmad A Menezes J. Host’s innate immune response to fungal and bacterial agents in *vitro*: up-regulation of interleukin-15 gene expression resulting in enhanced natural killer cell activity. Immunology. (2003) 109:263–70. doi: 10.1046/j.1365-2567.2003.01659.x PMC1782963 12757622

[74] Rose-John S Winthrop K Calabrese L. The role of IL-6 in host defence against infections: immunobiology and clinical implications. Nat Rev Rheumatol. (2017) 13:399–409. doi: 10.1038/nrrheum.2017.83 28615731

