# Supplementary material for: The effect of infection with the entomopathogenic fungus Conidiobolus coronatus (Entomopthorales) on eighteen cytokine-like proteins in Galleria mellonella (Lepidoptera) larvae
Source: Front Immunol. 2024 May 7;15:1385863. doi: 10.3389/fimmu.2024.1385863 (PMC11106378; doi:10.3389/fimmu.2024.1385863)
Supplement: Supplementary file 1 [file DataSheet_1.pdf]

Figure S1. Preliminary proteomic analysis, the human amino acid sequences of the 18 studied cytokines (IL-1α, IL-1β, IL-2, IL-3, IL-6, IL-7, IL-8, IL-12, IL-13, IL-15, IL-17, IL-19, IFN-γ, TNF-α, TNF-β, GM-CSF, M-CSF, G-CSF) compared with the *G. mellonella* proteomic database.

Sequences of human cytokines acquired from UniProt [https://doi.org/10.1093/nar/gkac1052] where used as queries in blastp searches (BLASTP 2.12.0+) against UniProtKB reference genomes + Swiss-Prot databases with results restricted to *G. mellonella* (taxon ID 7137).

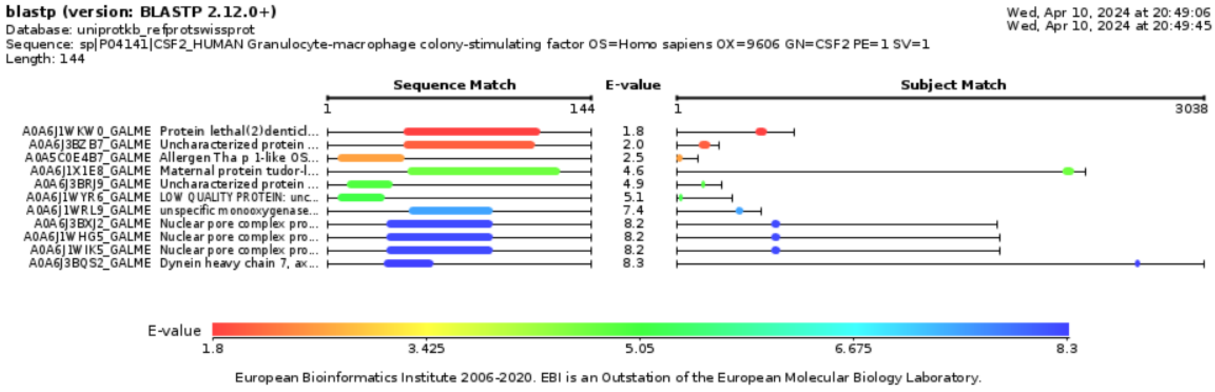

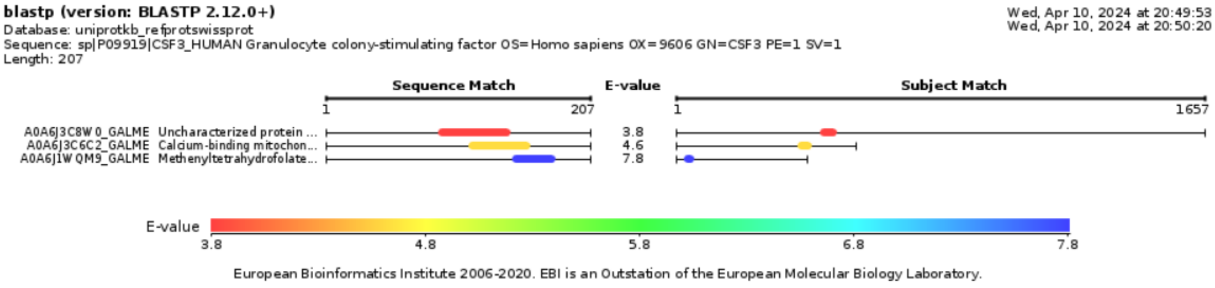

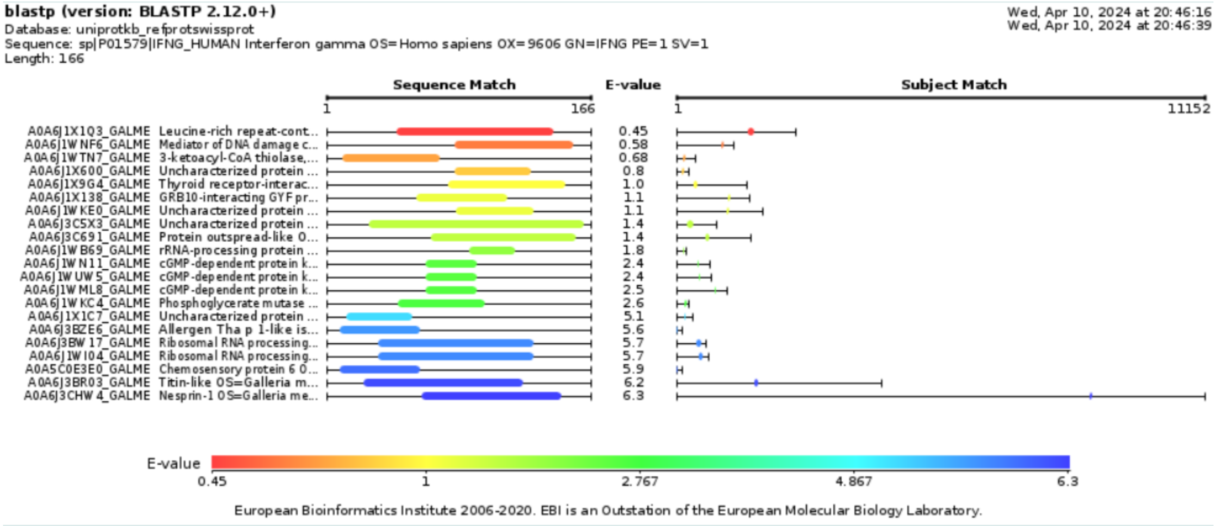

blastp (version: BLASTP 2.12.0+)
Database: uniprotkb_refprotswissprot
Sequence: sp|P01583|IL1A_HUMAN Interleukin-1 alpha OS=Homo sapiens OX=9606 GN=IL1A PE=1 SV=1
Length: 271

Wed, Apr 10, 2024 at 15:36:20
Wed, Apr 10, 2024 at 15:36:59

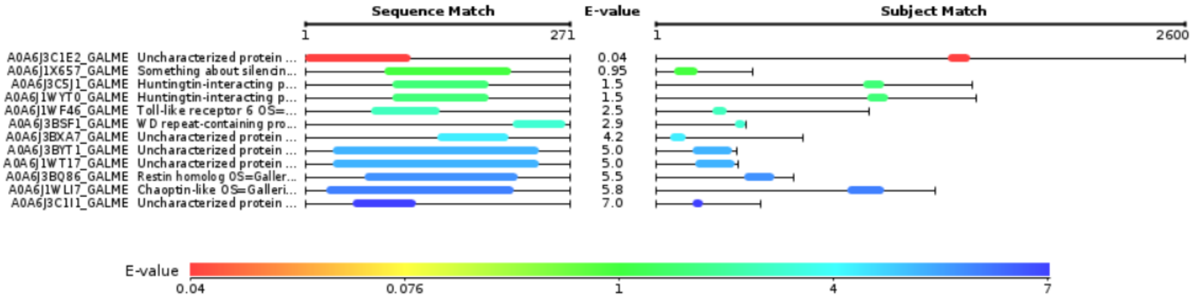

European Bioinformatics Institute 2006-2020. EBI is an Outstation of the European Molecular Biology Laboratory.

blastp (version: BLASTP 2.12.0+)
Database: uniprotkb_refprotswissprot
Sequence: sp|P01584|IL1B_HUMAN Interleukin-1 beta OS=Homo sapiens OX=9606 GN=IL1B PE=1 SV=2
Length: 269

Wed, Apr 10, 2024 at 16:20:03
Wed, Apr 10, 2024 at 16:21:18

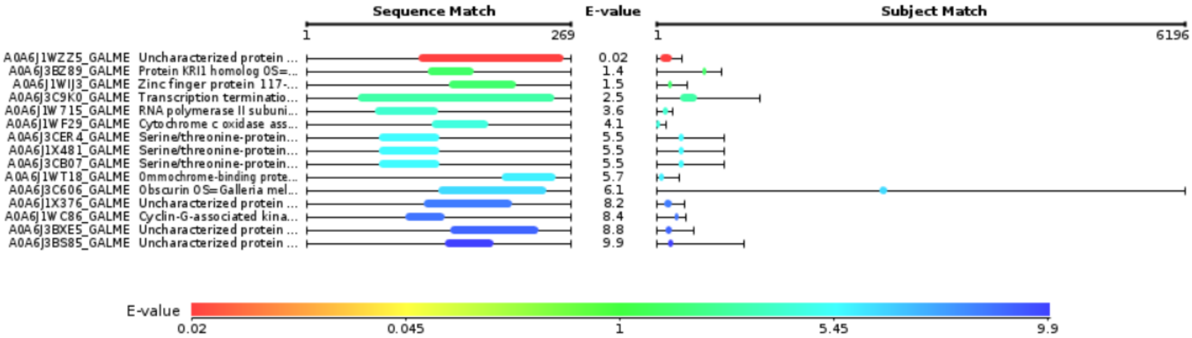

European Bioinformatics Institute 2006-2020. EBI is an Outstation of the European Molecular Biology Laboratory.

blastp (version: BLASTP 2.12.0+)
Database: uniprotkb_refprotswissprot
Sequence: sp|P08700|IL3_HUMAN Interleukin-3 OS=Homo sapiens OX=9606 GN=IL3 PE=1 SV=2
Length: 152

Wed, Apr 10, 2024 at 20:20:24
Wed, Apr 10, 2024 at 20:20:50

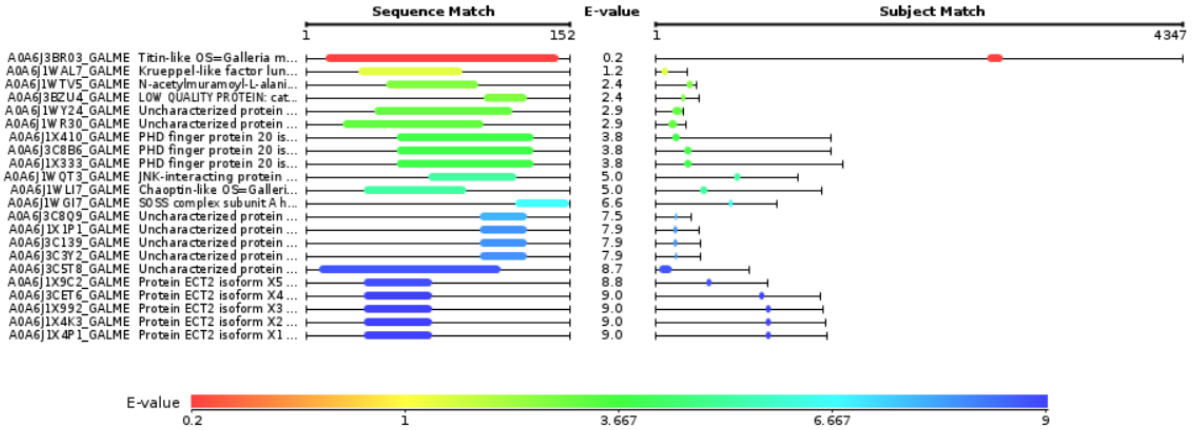

European Bioinformatics Institute 2006-2020. EBI is an Outstation of the European Molecular Biology Laboratory.

blastp (version: BLASTP 2.12.0+)
Database: uniprotkb_refprotswissprot
Sequence: sp|P60568|IL2_HUMAN Interleukin-2 OS=Homo sapiens OX=9606 GN=IL2 PE=1 SV=1
Length: 153

Wed, Apr 10, 2024 at 20:14:18
Wed, Apr 10, 2024 at 20:14:48

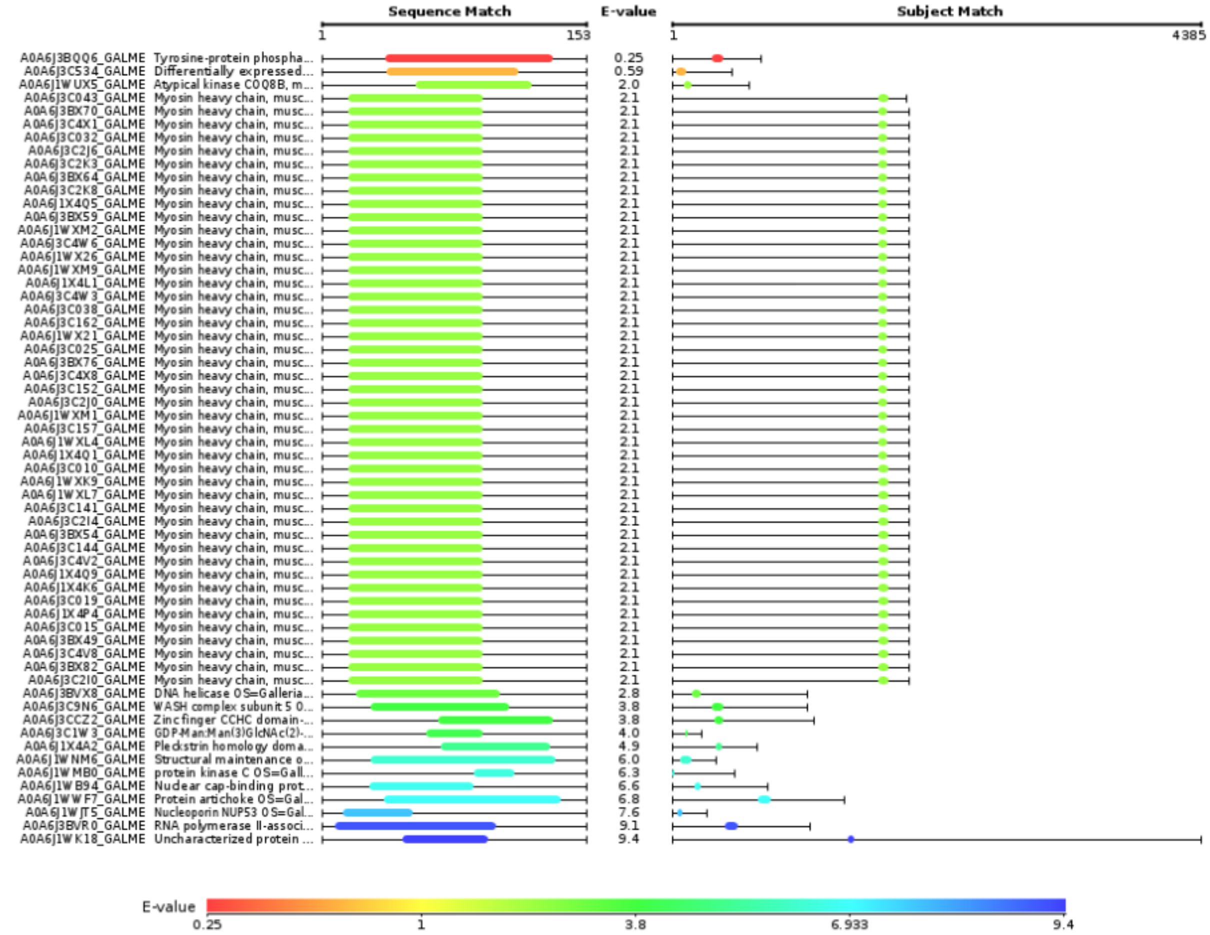

European Bioinformatics Institute 2006-2020. EBI is an Outstation of the European Molecular Biology Laboratory.

blastp (version: BLASTP 2.12.0+)
Database: uniprotkb_refprotswissprot
Sequence: sp|P13232|IL7_HUMAN Interleukin-7 OS=Homo sapiens OX=9606 GN=IL7 PE=1 SV=1
Length: 177

Wed, Apr 10, 2024 at 20:31:49
Wed, Apr 10, 2024 at 20:32:35

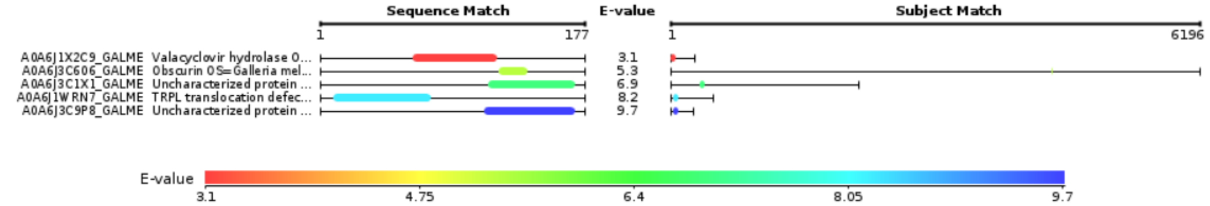

European Bioinformatics Institute 2006-2020. EBI is an Outstation of the European Molecular Biology Laboratory.

blastp (version: BLASTP 2.12.0+)
Database: uniprotkb_refprotswissprot
Sequence: sp|P10145|IL8_HUMAN Interleukin-8 OS=Homo sapiens OX=9606 GN=CXCL8 PE=1 SV=1
Length: 99

Wed, Apr 10, 2024 at 20:33:51
Wed, Apr 10, 2024 at 20:34:17

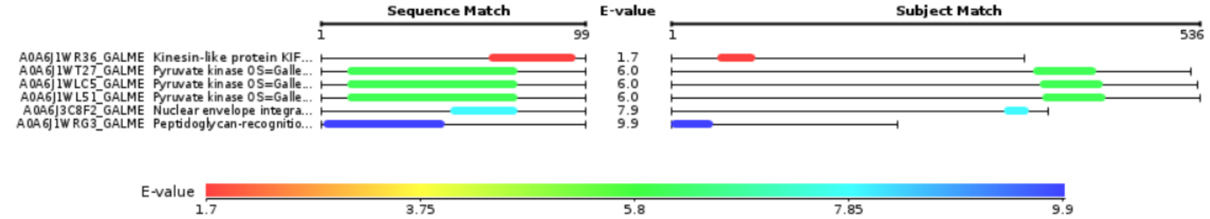

European Bioinformatics Institute 2006-2020. EBI is an Outstation of the European Molecular Biology Laboratory.

blastp (version: BLASTP 2.12.0+)
Database: uniprotkb_refprotswissprot
Sequence: sp|P05231|IL6_HUMAN Interleukin-6 OS=Homo sapiens OX=9606 GN=IL6 PE=1 SV=1
Length: 212

Wed, Apr 10, 2024 at 20:27:20
Wed, Apr 10, 2024 at 20:27:45

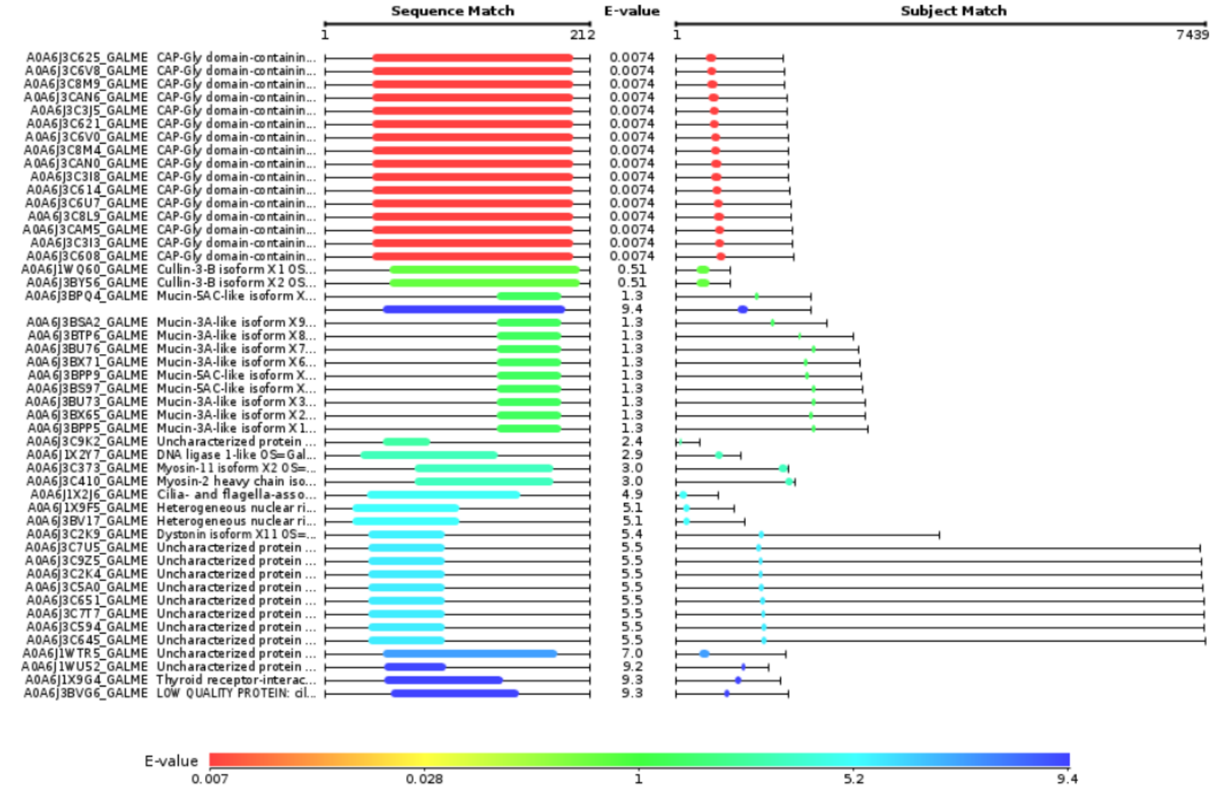

European Bioinformatics Institute 2006-2020. EBI is an Outstation of the European Molecular Biology Laboratory.

blastp (version: BLASTP 2.12.0+)
Database: uniprotkb_refprotswissprot
Sequence: sp|P29459|IL12A_HUMAN Interleukin-12 subunit alpha OS=Homo sapiens OX=9606 GN=IL12A PE=1 SV=2
Length: 219

Wed, Apr 10, 2024 at 20:35:57
Wed, Apr 10, 2024 at 20:36:34

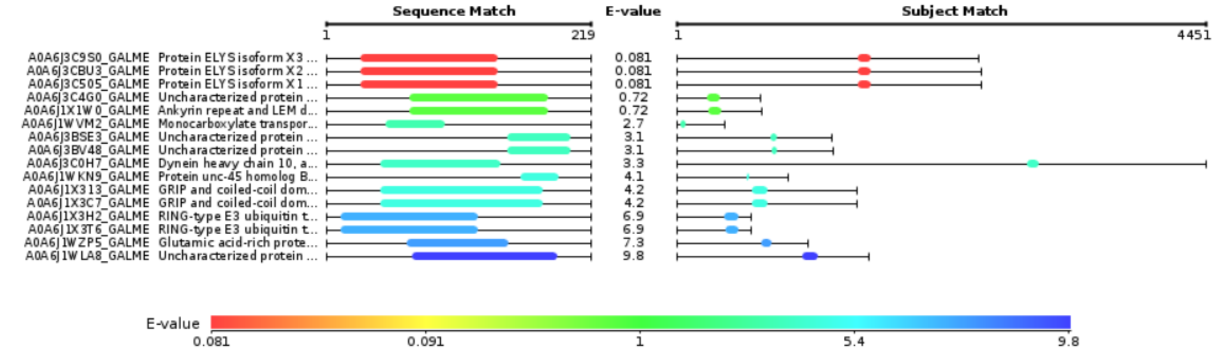

European Bioinformatics Institute 2006-2020. EBI is an Outstation of the European Molecular Biology Laboratory.

blastp (version: BLASTP 2.12.0+)
Database: uniprotkb_refprotswissprot
Sequence: sp|P35225|IL13_HUMAN Interleukin-13 OS=Homo sapiens OX=9606 GN=IL13 PE=1 SV=3
Length: 146

Wed, Apr 10, 2024 at 20:41:29
Wed, Apr 10, 2024 at 20:41:59

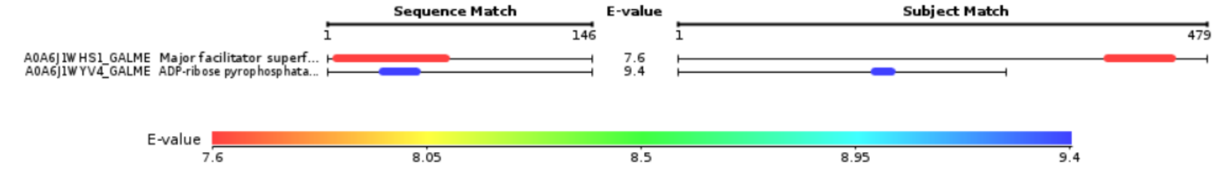

European Bioinformatics Institute 2006-2020. EBI is an Outstation of the European Molecular Biology Laboratory.

blastp (version: BLASTP 2.12.0+)

Database: uniprotkb_refprotswissprot
Sequence: sp|P29460|IL12B_HUMAN Interleukin-12 subunit beta OS=Homo sapiens OX=9606 GN=IL12B PE=1 SV=1
Length: 328

Wed, Apr 10, 2024 at 20:39:51
Wed, Apr 10, 2024 at 20:40:16

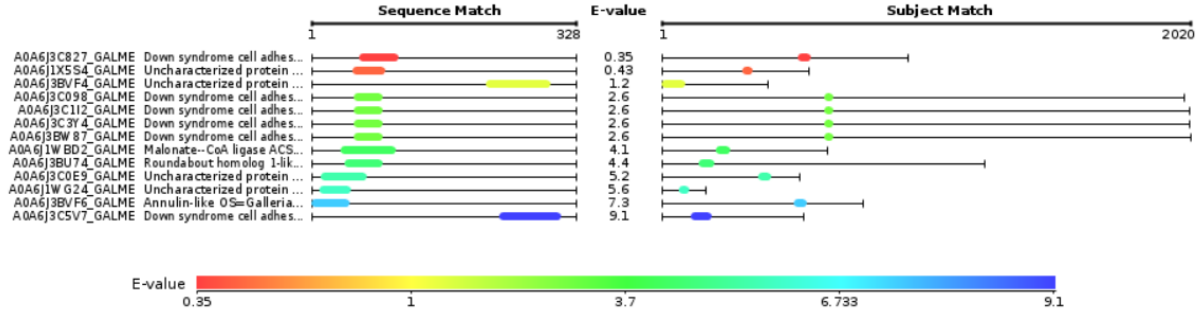

European Bioinformatics Institute 2006-2020. EBI is an Outstation of the European Molecular Biology Laboratory.

blastp (version: BLASTP 2.12.0+)

Database: uniprotkb_refprotswissprot
Sequence: sp|P40933|IL15_HUMAN Interleukin-15 OS=Homo sapiens OX=9606 GN=IL15 PE=1 SV=1
Length: 162

Wed, Apr 10, 2024 at 20:41:56
Wed, Apr 10, 2024 at 20:42:27

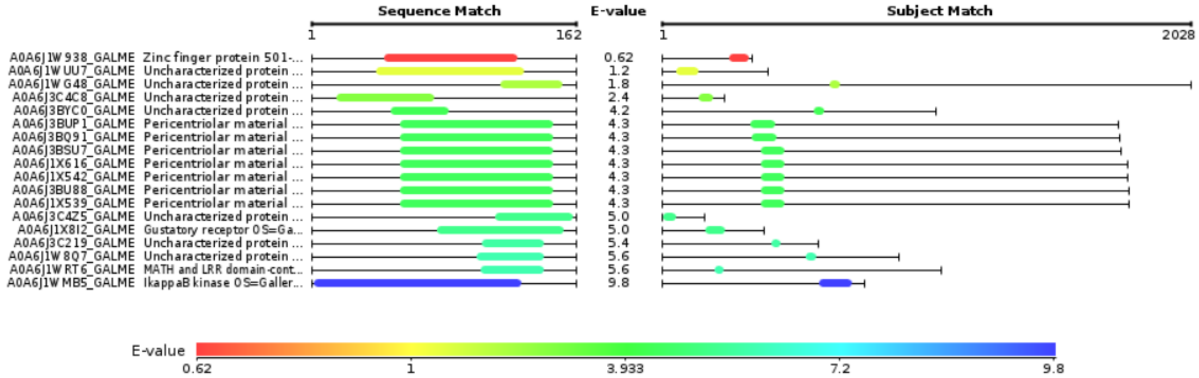

European Bioinformatics Institute 2006-2020. EBI is an Outstation of the European Molecular Biology Laboratory.

blastp (version: BLASTP 2.12.0+)

Database: uniprotkb_refprotswissprot
Sequence: sp|Q16552|IL17_HUMAN Interleukin-17A OS=Homo sapiens OX=9606 GN=IL17A PE=1 SV=1
Length: 155

Wed, Apr 10, 2024 at 20:43:14
Wed, Apr 10, 2024 at 20:43:43

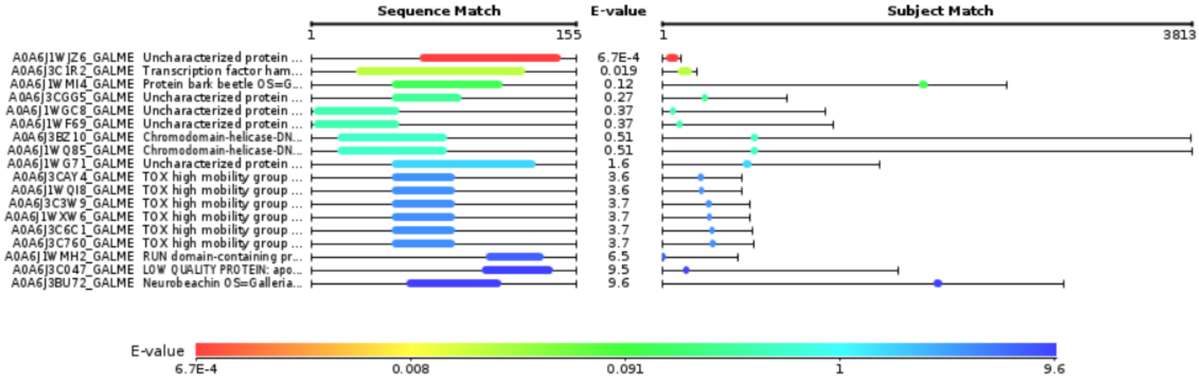

European Bioinformatics Institute 2006-2020. EBI is an Outstation of the European Molecular Biology Laboratory.

blastp (version: BLASTP 2.12.0+)
Database: uniprotkb_refprotswissprot
Sequence: sp|Q9UHF5|IL17B_HUMAN Interleukin-17B OS=Homo sapiens OX=9606 GN=IL17B PE=2 SV=1
Length: 180

Wed, Apr 10, 2024 at 21:16:54
Wed, Apr 10, 2024 at 21:17:19

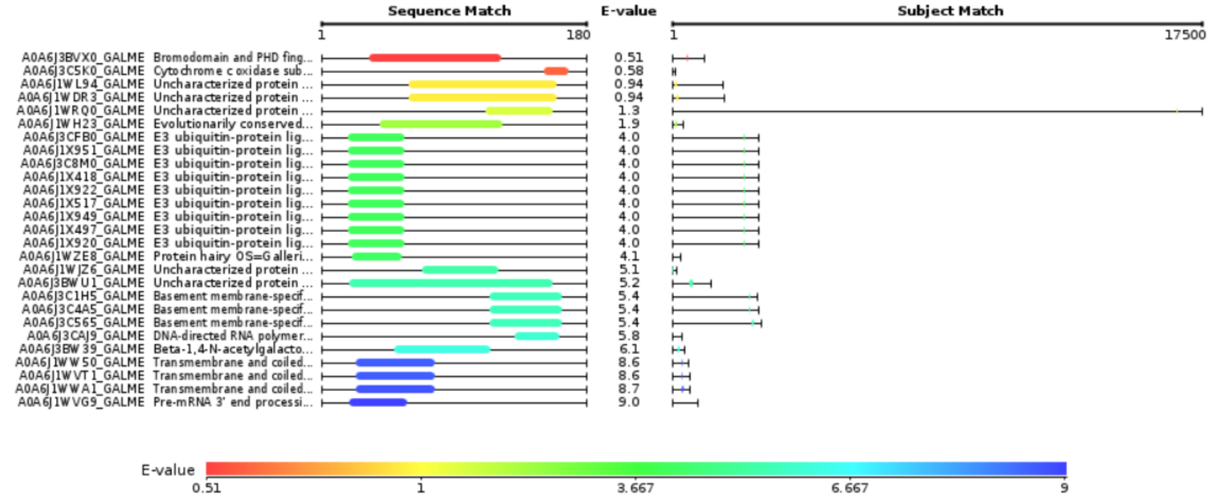

European Bioinformatics Institute 2006-2020. EBI is an Outstation of the European Molecular Biology Laboratory.

blastp (version: BLASTP 2.12.0+)
Database: uniprotkb_refprotswissprot
Sequence: sp|Q9UHD0|IL19_HUMAN Interleukin-19 OS=Homo sapiens OX=9606 GN=IL19 PE=1 SV=2
Length: 177

Wed, Apr 10, 2024 at 20:45:42
Wed, Apr 10, 2024 at 20:46:11

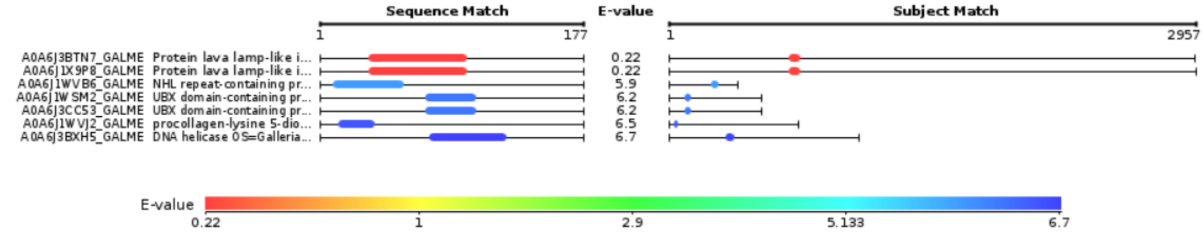

European Bioinformatics Institute 2006-2020. EBI is an Outstation of the European Molecular Biology Laboratory.

blastp (version: BLASTP 2.12.0+)
Database: uniprotkb_refprotswissprot
Sequence: sp|P01375|TNFA_HUMAN Tumor necrosis factor OS=Homo sapiens OX=9606 GN=TNF PE=1 SV=1
Length: 233

Wed, Apr 10, 2024 at 20:47:13
Wed, Apr 10, 2024 at 20:47:37

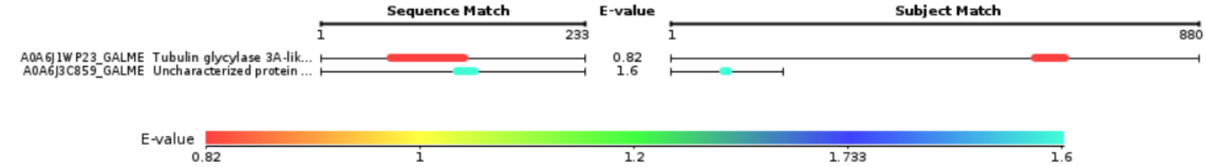

European Bioinformatics Institute 2006-2020. EBI is an Outstation of the European Molecular Biology Laboratory.

blastp (version: BLASTP 2.12.0+)
Database: uniprotkb_refprotswissprot
Sequence: sp|P01374|TNFB_HUMAN Lymphotoxin-alpha OS=Homo sapiens OX=9606 GN=LTA PE=1 SV=2
Length: 205

Wed, Apr 10, 2024 at 20:48:51
Wed, Apr 10, 2024 at 20:49:23

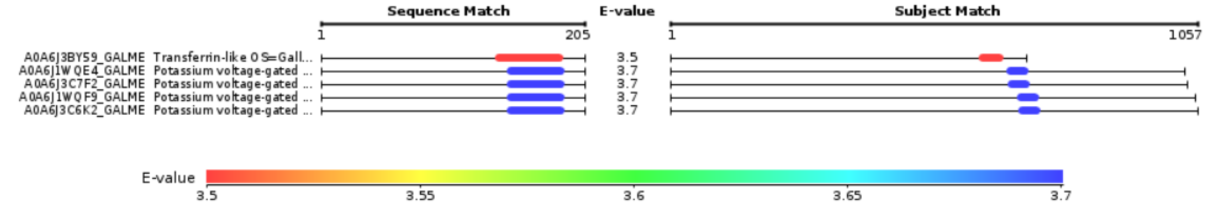

European Bioinformatics Institute 2006-2020. EBI is an Outstation of the European Molecular Biology Laboratory.

blastp (version: BLASTP 2.12.0+)
Database: uniprotkb_refprotswissprot
Sequence: sp|P09603|CSF1_HUMAN Macrophage colony-stimulating factor 1 OS=Homo sapiens OX=9606 GN=CSF1 PE=1 SV=2
Length: 554

Wed, Apr 10, 2024 at 20:50:46
Wed, Apr 10, 2024 at 20:51:18

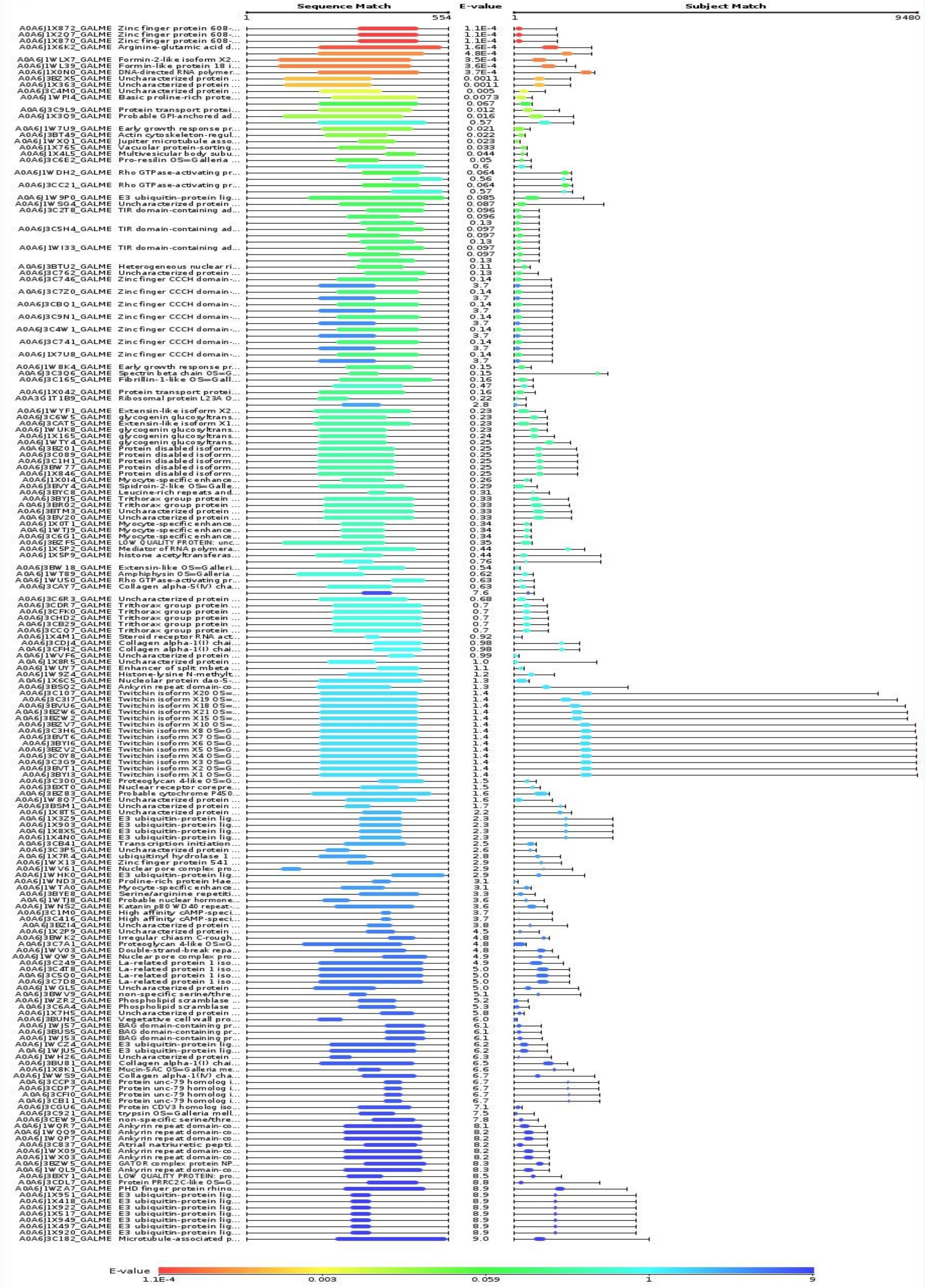

European Bioinformatics Institute 2006-2020. EBI is an Outstation of the European Molecular Biology Laboratory.
